# Silicon carbonate, Si[CO_3_]_2_, is a potential carbon host in the deep Earth

**DOI:** 10.1126/sciadv.aee5766

**Published:** 2026-05-29

**Authors:** Dominik Spahr, Lkhamsuren Bayarjargal, Valentin Kovalev, Ninel Sharapova, Lukas Brüning, Pascal L. Jurzick, Sean S. Sebastian, Maxim Bykov, Victor Milman, Konstantin Glazyrin, Elena Bykova, Björn Winkler

**Affiliations:** ^1^ Institute of Geosciences, Goethe University Frankfurt, Altenhöferallee 1, 60438 Frankfurt, Germany.; ^2^ Department of Geosciences, Stony Brook University, 255 Earth & Space Science Building, Stony Brook, NY 11794-2100, USA.; ^3^ Institute of Inorganic and Analytical Chemistry, Goethe University Frankfurt, Max-von-Laue-Straße 7, 60438 Frankfurt, Germany.; ^4^ Dassault Systèmes BIOVIA, 22 Cambridge Science Park, Cambridge CB4 0FJ, UK.; ^5^ Deutsches Elektronen-Synchrotron DESY, Notkestrasse 85, 22607 Hamburg, Germany.

## Abstract

The transport of carbon into the deep Earth is governed by the stability and properties of carbon-bearing phases. However, despite extensive research efforts, it is still an open question whether there are high-pressure minerals that can incorporate both silicon and carbon simultaneously. Multiple theoretical studies suggest that Si─C─O compounds could be stabilized at high pressures, but so far, no reliable experimental evidence for their presence has been presented. Here, we demonstrate that at 40(2) gigapascals and ≈ 1800(200) kelvin, CO_2_ reacts with silicic acid or cristobalite and forms the anhydrous silicon carbonate Si[CO_3_]_2_. The structure consists of [SiO_6_] octahedra coordinated by six [CO_3_]^2−^ groups. Similar groups occur in many ambient and high-pressure carbonates, suggesting that mixed carbonate-silicate phases may be stable at midmantle pressures. Silicon carbonate decomposes upon decompression at pressures of <6 gigapascals but could act as a potential host for carbon in Earth’s lower mantle.

## Teaser

Synthesizing a carbonate of silicon at the *p*,*T* conditions of Earth’s lower mantle.

## INTRODUCTION

Carbonates, the main reservoir for carbon in Earth’s crust, are transported into the deep Earth by subduction of oceanic lithosphere (*1*–*3*). The total amount of carbon transported into Earth’s mantle is roughly 40 to 60 Mt per year (*4*, *5*). Hence, a prerequisite for understanding the global carbon cycle is to determine structures, stabilities, and properties of high-pressure carbon-bearing phases. The chemically simple anhydrous carbonates Ca[CO_3_] and Ca,Mg[CO_3_]_2_ account for >90% of the carbonates in Earth’s crust (*1*). Consequently, their *p*,*T* phase diagrams have been studied extensively in the last decades and there is a consensus of phase stabilities in these end-member systems (*6*, *7*). Multiple studies suggest that carbonates break down during subduction (*8*–*10*), and hence, it is of interest to understand the stabilities and reactions of carbonates and minerals typical for a pyrolitic mantle at high CO_2_ fugacities. At high CO_2_ fugacities and moderate pressures, anhydrous inorganic pyrocarbonates may form (*11*–*13*), and it has long been debated if reactions of carbonates with a pyrolitic mantle would lead to the formation of a silicon carbonate.

However, up to now, no unambiguous evidence, i.e., a reliable structural model derived from a diffraction study, has been presented for a silicon carbonate yet. A previous study investigated the reaction between carbon dioxide and silica at 18 to 26 GPa and 296 to 980 K (*14*). On the basis of Raman and infrared spectroscopy and synchrotron powder x-ray diffraction, it was claimed that one or more silicon carbonates of unknown composition ${\text{Si}}_{x}C_{y}O_{2x+2y}$ had been obtained. However, that study provided neither lattice parameters, nor a structural model, nor unambiguous spectroscopic evidence, such as a well-defined Raman band due to an expected [CO_3_]^2−^ stretching vibration. A further experimental study of the system SiO_2_-CO_2_ (*15*) was later retracted (*16*). To facilitate a formation reaction, CO_2_-filled SiO_2_ zeolite has been studied at high pressures (≈24 GPa) and temperatures (up to 825 K), but no chemical reaction was observed (*17*).

In addition to the experimental studies, several crystal structure prediction approaches have been used to find stable phases in the Si─C─O system (*18*–*22*). Morales-García *et al.* (*18*) concluded that up to 30 GPa, all investigated compounds with composition ${\text{SiC}}_{x}O_{y}$ were thermodynamically unstable, but that the least unstable compound was a monoclinic polymorph of ${\text{SiC}}_{2}O_{6}$. Zhou *et al.* (*19*) predicted the formation of a compound which crystallizes in a trigonal space group with ${\text{SiC}}_{2}O_{6}$ composition and conjectured that it may be synthesized through a reaction of SiO_2_ and CO_2_ at 20 GPa at low temperature. Marqués *et al.* (*20*) predicted a monoclinic structure with ${\text{SiC}}_{2}O_{6}$ stoichiometry in a pressure range of 7.2 to 41 GPa. Qu *et al.* (*21*) deduced from ab initio molecular dynamics (MD) simulations that CO_2_ would react with SiO_2_ and would form amorphous compounds at 43 GPa and 800 K. Yong *et al.* (*22*) proposed from ab initio MD simulations that a CO_2_-SiO_2_ solid solution would be formed and be stable above 10 GPa. Clearly, these modeling results are mutually inconsistent and none of them are supported by experiments, e.g., by comparison of a computed vibrational spectrum to an experimentally measured one, let alone by a comparison of a structure derived from diffraction data to a structural model from modeling.

In this study, we successfully synthesized silicon carbonate, Si[CO_3_]_2_, in laser-heated diamond-anvil cells (LH-DACs) and characterized its crystal structure using single-crystal x-ray microdiffraction, Raman spectroscopy, and density functional theory (DFT) calculations. We show that silicon carbonate can form at 40 to 45 GPa and ≈1800 K through a reaction between silicic acid [SiO*_x_*(OH)_4-2*x*_] and CO_2_ or between cristobalite (SiO_2_) and CO_2_. These *p*,*T* conditions correspond to depths of 1000 to 1200 km beneath Earth’s surface (*23*–*25*). We chose silicic acid as the starting material in one set of experiments because hydrous silicic fluids are often observed in diamond inclusions (*26*). The analogous reaction with cristobalite were carried out to test whether Si[CO_3_]_2_ would also be formed in a “dry” environment. Silicon carbonate remains stable upon decompression to at least 6 GPa at ambient temperature and might be a possible host of carbon in Earth’s lower mantle.

## RESULTS

**Fig. 1.**
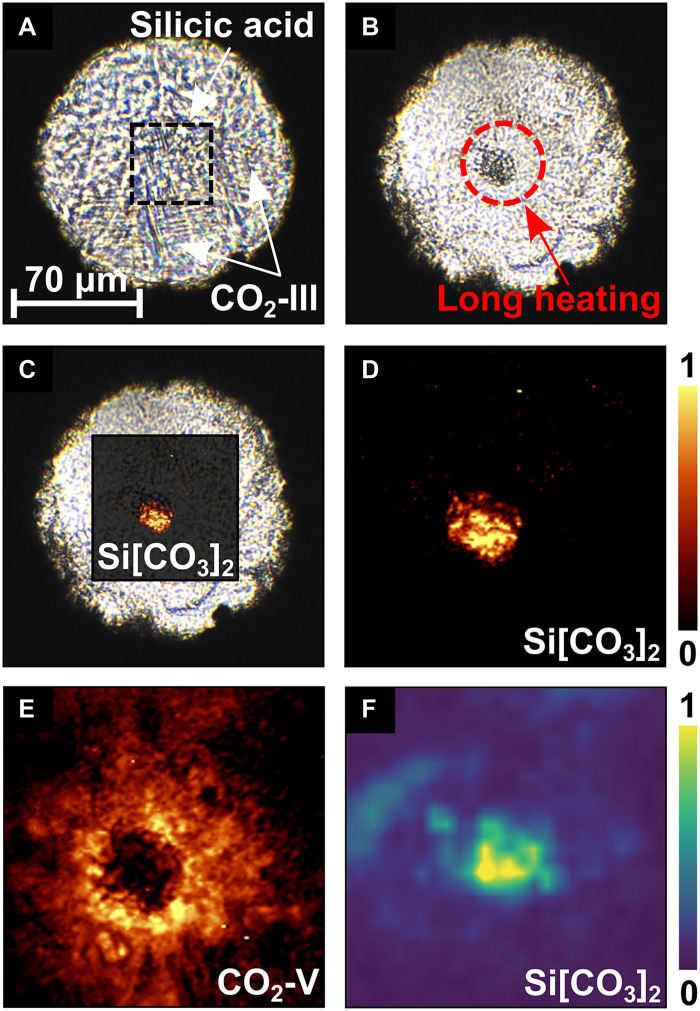
Synthesis of silicon carbonate at 40(2) GPa. (**A**) Sample chamber of the DAC with the unheated silicic acid + CO_2_ at 30(2) GPa. (**B**) Silicic acid + CO_2_ after laser heating to ≈1800(200) K at 40(2) GPa. (**C**) Raman map of Si[CO_3_]_2_-*P*2_1_/*n* after the synthesis overlaid on a picture of the sample chamber. (**D**) Raman map of Si[CO_3_]_2_-*P*2_1_/*n* ($\approx 630\;{\text{cm}}^{–1}$). (**E**) Raman map of CO_2_-V ($\approx 805\;{\text{cm}}^{–1}$). (**F**) XRD map of Si[CO_3_]_2_-*P*2_1_/*n*, where the intensity of the reflections at $2\theta \approx 4.6^{\circ }$ is plotted.

**Fig. 2.**
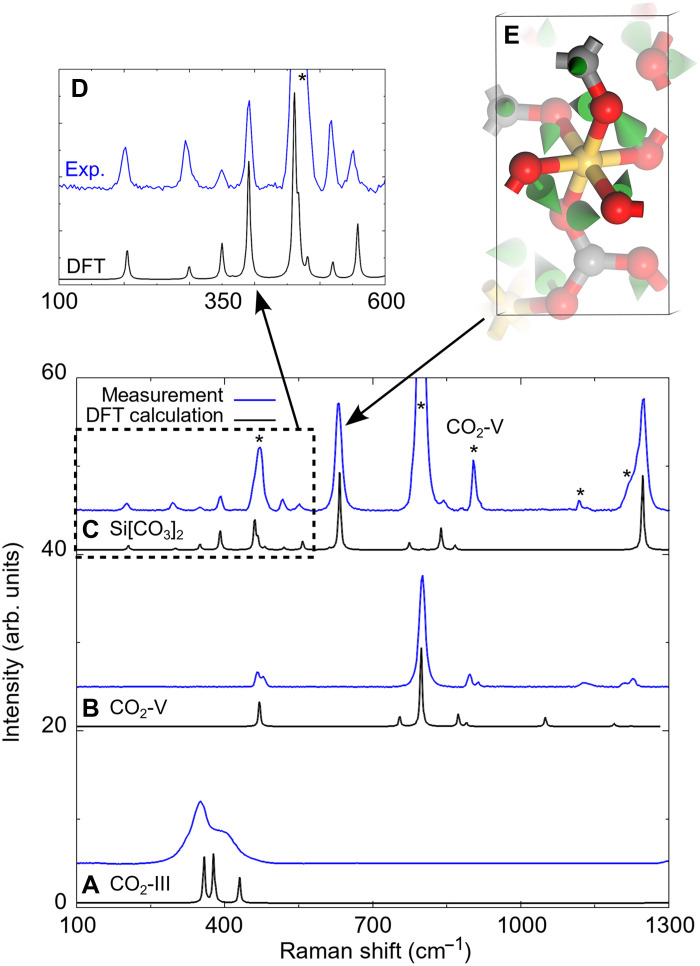
Raman spectroscopy at 40(2) GPa. (**A**) Raman spectra for CO_2_-III before the laser heating. (**B**) Raman spectra for CO_2_-V GPa after the laser-heating. (**C**) Raman spectra of Si[CO_3_]_2_-*P*2_1_/*n* after the synthesis. (**D**) Enlargement of the frequency range between 100 and 600 cm^−1^. Experimental Raman spectra are shown in blue, and DFT-based calculations are shown in black. The Raman shifts of the theoretical spectra were scaled by a single factor of 2 to 4% to facilitate a comparison to the experimental data. This scaling effectively accounts for the well-established GGA-PBE underbinding. Peaks of CO_2_-V in the Raman spectrum of Si[CO_3_]_2_-*P*2_1_/*n* are marked by an asterisk (∗). (**E**) Eigenvector of the atomic displacements for the characteristic Raman mode of Si[CO_3_]_2_-*P*2_1_/*n* at $\approx 630\;{\text{cm}}^{–1}$.

High-pressure experiments attempting the synthesis of silicon carbonate were carried out using LH-DACs between 30 and 45 GPa. Figure 1A shows the sample chamber of the DAC after the loading and compressing the DAC to 30(2) GPa. Silicon carbonate was first synthesized from a mixture of silicic acid and CO_2_ that was compressed in a DAC to 40(2) GPa and then laser heated to 1800(200) K for 60 min to promote a reaction toward thermodynamically stable phases (Fig. 1B). Spatially resolved Raman spectroscopy shows that the novel phase forms at the center of the heated area (Fig. 1, C and D), evidenced by the appearance of strong Raman modes at 630 and 1250 cm^−1^ (Fig. 2C), whereas the initial amorphous silicic acid produces no detectable Raman signal. Unreacted carbon dioxide fully transforms to the CO_2_-V polymorph, which is stable at these *p*,*T* conditions (*27*, *28*), and is characterized by a strong Raman mode at $\approx 805\;{\text{cm}}^{–1}$ (Fig. 2B). Before the laser heating, CO_2_-III was present across the sample chamber (Fig. 2A), which remains meta-stable up to very high pressures without heating (*27*, *29*, *30*). The experimental spectra of the CO_2_ polymorphs III and V are in good agreement with the one derived from our DFT-based calculations (Fig. 2, A and B). Similar experiments at lower pressures suggest that the same silicon carbonate phase forms between 35(2) and 45(2) GPa; at 30(2) GPa, no new Raman modes were observed at $\approx 630\;{\text{cm}}^{–1}$ or at $\approx 1250\;{\text{cm}}^{–1}$, while at 35(2) GPa, weak peaks appeared. A Raman mode occurring at $\approx 1250\;{\text{cm}}^{–1}$ at this pressure could be indicative for the presence of a *sp*^2^ carbonate. For example, in Mg[CO_3_] (50 GPa) or for Be[CO_3_] (60 GPa), the [CO_3_]^2−^ stretching mode had been observed at $\approx 1200\;{\text{cm}}^{–1}$ (*31*, *32*). However, it is well established that even for isostructural carbonates, the position of the [CO_3_]^2−^ stretching mode changes substantially with pressure, the size of the cation, and across phase transitions (*6*, *31*–*33*).

**Fig. 3.**
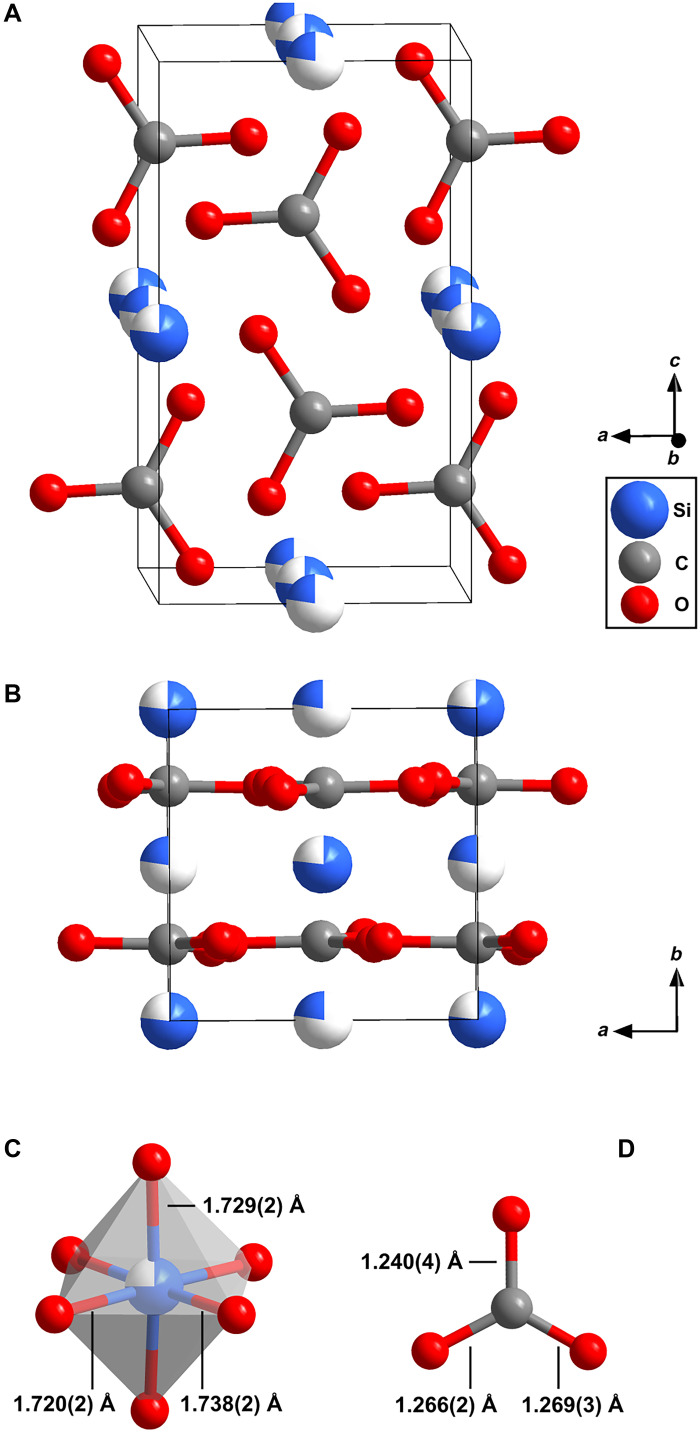
Crystal structure of Si[CO_3_]_2_ obtained from synchrotron single-crystal x-ray diffraction at 40(2) GPa. The experimental crystal structure of Si[CO_3_]_2_ (*P*2_1_/*n*, *Z* = 4) is shown (**A**) along the *b* axis and (**B**) along the *c* axis. The positions of the silicon atoms are partially occupied. (**C**) A [SiO_6_] octahedron with selected Si─O bond distances. (**D**) Geometry of the symmetrically independent [CO_3_]^2−^ group with selected C─O bond distances.

X-ray diffraction (XRD) was carried out to determine the chemical composition and crystal structure of the unknown silicon carbonate phase. First, we collected XRD data on a two-dimensional grid across the area where we had located the unknown phase by Raman spectroscopy. The XRD map of the reflections at $2\theta \approx 4.6^{\circ }$ (Fig. 1F) are in good agreement with the Raman map (Fig. 1, C and D) of the unknown phase. Hence, we collected diffraction data suitable for single crystal XRD on grid positions where unidentified reflections were present at $2\theta \approx 4.6^{\circ }$. We solved the crystal structure of the unknown phase at 40(2) GPa and found that it is a silicon *sp*^2^ carbonate with Si[CO_3_]_2_ composition. It crystallizes in the monoclinic space group *P*2_1_/*n* with *Z* = 4 (Fig. 3). The lattice parameters at 40(2) GPa are a=4.141(2) Å, b=4.1916(9) Å, c=7.2283(8) Å, and $\beta =89.88{\left(2\right)}^{\circ }$ [$V=125.47\left(6\right)\;{Å}^{3}$]. The structural data are listed in tables S1 and S2. The reflection intensity distribution visualized in the XRD map (Fig. 1H) belongs to a combination of the ($0\bar{1}1$), (002), (101), and ($\bar{1}01$) lattice planes of Si[CO_3_]_2_-*P*2_1_/*n*.

In the crystal structure of Si[CO_3_]_2_-*P*2_1_/*n*, two Wyckoff positions (2*d* at $0,\frac{1}{2},\frac{1}{2}$ and 2*b* at $0,0,\frac{1}{2}$) are occupied by silicon. An unconstrained refinement of the occupancy resulted in a total silicon content of ≈1 per formula unit, with an approximate occupation of 0.75 and 0.25 for site 2*b* and site 2*d*, respectively. This is consistent with the charge balance of one Si^4+^ cation by two [CO_3_]^2−^ groups. A refinement with a full silicon occupancy of the 2*b* position resulted in a strong increase of *R*_1_ value from 4.1 to 20%. In addition, we solved the crystal structure of another Si[CO_3_]_2_-*P*2_1_/*n* crystal in the sample chamber of the DAC at the same pressure. The atomic positions after both structure refinements are identical within the experimental uncertainties. However, the silicon occupancy between the two positions is substantially different, as in the second crystal, and it was 0.95 and 0.05 for the two sites. Again, a refinement with a full silicon occupancy of the 2*b* position resulted in a substantially increased *R*_1_ value (from 5 to 7%).

To understand the role of the partial site occupancies in the experimental structural models, we carried out DFT-based full geometry optimizations. For the DFT calculations, we fully occupied the 2*b* site and left the other site (2*d*) empty. This allows to use a cell with the same volume ($\approx 125\;{Å}^{3}$) than the experimental structural model. The theoretical Raman spectrum derived from this DFT calculation reproduces the experimental Raman spectrum very well (Fig. 2, C and D). In the second step, we performed a DFT-based full geometry optimization with an inversion of the site occupancies and fully occupied the 2*d* site. Our analysis showed that an inversion of the site occupancies leads to the same structure, which is related to the first structure by a shift of origin only. Hence, structures with partial site occupancies can be viewed as a combination of two identical structures. The partial site occupancies describe an average occupancy. If a specific site in a specific unit cell is occupied, the likelihood that the close neighbor is occupied is very small. In the next unit cell, the occupancies may be reversed. In the averaged structural model, it then looks like two silicon atoms may be very close to each other, but due to short-range ordering, this is not the case. Raman spectroscopy is a local probe, and as the local environment of the Si atoms is the same for the two sites, Raman spectroscopy is not sensitive to the site occupancies in the present case. We analyzed the eigenvectors of some modes. The dominant Raman mode at $\approx 630\;{\text{cm}}^{-1}$ belongs to a complex distortion of the [SiO_6_] octahedra (Fig. 2E). In addition, our analysis of the eigenvectors clearly shows that the Raman mode at $\approx 1250\;{\text{cm}}^{-1}$ belongs to a [CO_3_]^2−^ stretching vibration (fig. S3).

The crystal structure of Si[CO_3_]_2_-*P*2_1_/*n* obtained here experimentally is closely related to the results obtained from a crystal structure prediction study by Marqués *et al.* (*20*). While the positions of the carbon and oxygen atoms agree with each other, in the crystal structure prediction study, the silicon atom was only located on one position and no partial site occupancies were reported. The one reported position for the silicon atoms agrees with one of the positions in our experimental data, while the second position was unoccupied. Marqués *et al.* (*20*) also suggested that the stability field of Si[CO_3_]_2_-*P*2_1_/*n* ranges from about 7.2 to 41 GPa, whereas in our experiments, the compound could not be synthesized below roughly 35 GPa. Silicon carbonate remains present upon decompression at ambient temperature to at least 6(1) GPa. Our Raman spectra (fig. S2) indicate a progressive deterioration of crystallinity during decompression, but bands attributed to the high-pressure phase are still clearly observable. Nevertheless the quality of the diffraction data collected at 6 GPa is reasonable and we were able to refine the data (table S1). On further decompression, a refinement of the data was not possible, but in the open DAC at 0 GPa, we could determine lattice parameters of Si[CO_3_]_2_-*P*2_1_/*n*. These are in reasonable agreement with those obtained from the DFT calculations. In the 0-GPa dataset, we could identify stishovite as a decomposition product. We find no evidence for carbon-silicon mixing on a single Wyckoff site. In contrast to the findings by Santoro *et al.* (*15*), our spectroscopic data clearly show the [CO_3_]^2−^ stretching vibration (Fig. 2C), confirming the presence of carbonate groups in the crystal structure. This is independently supported by DFPT calculations, which predict an intense C─O stretching mode (Fig. 2C). We therefore conclude that the absence of this spectroscopic feature is a strong indicator that [CO_3_]^2−^ groups are not present.

Si[CO_3_]_2_-*P*2_1_/*n* is an *sp*^2^ carbonate with nearly trigonal-planar [CO_3_]^2−^ groups as central building blocks (Fig. 3D). One symmetrically independent [CO_3_]^2−^ group is present in Si[CO_3_]_2_-*P*2_1_/*n*. The C─O bond distances within the [CO_3_]^2−^ group range from 1.240(4) to 1.269(3) Å and scatter around the values derived from our DFT calculations (1.261 to 1.265 Å). In addition, the range of experimental values for the O─C─O angles [119.0(1)° to 120.6(2)°] is slightly larger than the range of the values obtained from DFT (119.5° to 120.3°). The Mulliken population analysis of the C─O bonds in the [CO_3_]^2−^ groups yielded nearly identical values (0.89 and 0.90 e^−^/Å^3^). The geometry of the [CO_3_]^2−^ groups is in agreement with the geometry observed in other *sp*^2^ carbonates hosting small cations such as Be[CO_3_] or Mg[CO_3_] at elevated pressures (*32*, *34*, *35*). In the crystal structure of Si[CO_3_]_2_-*P*2_1_/*n*, the [CO_3_]^2−^ groups are arranged in layers parallel to the *b* axis.

The crystal structure of Si[CO_3_]_2_-*P*2_1_/*n* is also characterized by [SiO_6_] octahedra (Fig. 3C). The Si─O bond distances range from 1.720(2) to 1.738(2) Å at 40(2) GPa. The experimental Si─O bond distances are in agreement with our DFT-based calculations (1.71 to 1.73 Å). The DFT calculations reveal a predominantly covalent character of the Si─O bond with bond populations of 0.39 and 0.40 e^−^/Å^3^. The Si─O bond distances are in very good agreement with the ones observed in stishovite (SiO_2_-*P*4_2_/*mnm*) at the same pressure (≈1.71 Å) (*36*). Structural units of the type [*M*(CO_3_)_6_]^*n*–^, in which an octahedrally coordinated cation is surrounded by six parallel carbonate groups, occur in common carbonates such as calcite (Ca[CO_3_]), magnesite (Mg[CO_3_]), and siderite (Fe[CO_3_]), as well as in less common high-pressure Be-carbonate (Be[CO_3_]) and Al-carbonate (Al_2_[CO_3_]_3_) (*32*, *37*, *38*). These units accommodate a remarkably wide range of cation sizes within the [*M*O_6_] octahedron, from about 1.00 Å for Ca^2+^ down to about 0.54 Å for Al^3+^, 0.45 Å for Be^2+^, and 0.40 Å for Si^4+^ (*32*, *37*–*39*).

**Fig. 4.**
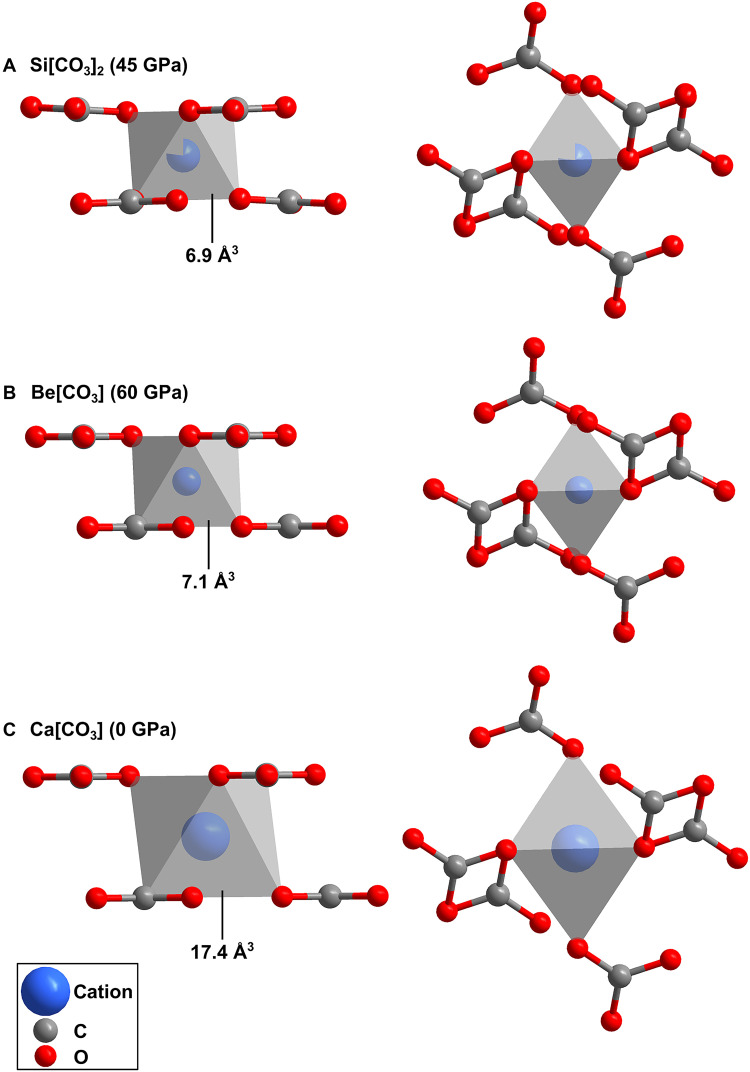
[*M*O_6_] octahedra coordinated by six parallel [CO_3_]^2−^ groups in different carbonates. (**A**) In Si[CO_3_]_2_-*P*2_1_/*n* at 40(2) GPa. (**B**) In Be[CO_3_]-$R\bar{3}c$ at 60(2) GPa (*32*). (**C**) In Ca[CO_3_]-$R\bar{3}c$ at ambient conditions (*37*).

Figure 4 shows a comparison of the [*M*O_6_] octahedra in Si[CO_3_]_2_ with the calcite-type carbonates Ca[CO_3_] and Be[CO_3_]. The [*M*O_6_] octahedra generally assemble into close-packed layers through shared carbonate groups: In calcite-type structures, the layers are flat and stacked in an ABCABC pattern (fig. S6), while in Al_2_[CO_3_]_3_, the layers are buckled and follow an ABAB sequence. In silicon carbonate, the layers are flat but stack in a single-layer AAA sequence (fig. S6), and partial Si occupancies help maintain charge balance and prevent formation of short Si-Si distances between [SiO_6_] octahedra from adjacent layers. The [SiO_6_] octahedra of the partially occupied silicon atoms share faces along the *b* axis (fig. S6). Along the *a* and *c* axes, the [SiO_6_] octahedra are connected by sharing a [CO_3_]^2−^ group. These structural similarities among carbonates of the most commonly encountered metal cations in the mantle (Mg, Ca, Fe, Si, and Al) suggest the possibility of forming solid solutions between end-members of high-pressure carbonate-silicate phases. Experimental and theoretical studies show that silicate melts can dissolve substantial amounts of CO_2_, with solubility increasing with pressure (*40*). Our observations indicate that [Si(CO_3_)_6_]^8−^ units can readily form under lower mantle conditions, suggesting that they may act as rigid structural fragments in models of carbonate-silicate melts in Earth’s deep interior.

Recently the crystal structure of U[CO_3_]_2_-*C*2, a chemically simple carbonate also hosting a tetravalent cation, has been described at 20(2) GPa (*41*). Similar to Si[CO_3_]_2_-*P*2_1_/*n*, the crystal structure of U[CO_3_]_2_-*C*2 is characterized by trigonal-planar [CO_3_]^2−^ groups. However, in U[CO_3_]_2_-*C*2, the [CO_3_]^2−^ groups are not oriented in layers. Instead, in a first approximation, they are randomly distributed around the uranium atoms. In addition, the uranium coordination by oxygen atoms is noticeably higher (10 and 12) and nearly spherical in contrast to the [SiO_6_] octahedra in Si[CO_3_]_2_-*P*2_1_/*n*. This is probably due to the much larger ionic radius of tetravalent uranium cations (0.89 Å in sixfold coordination) in contrast to the small silicon cations (0.40 Å) (*39*).

In a second set of experiments, synthetic cristobalite, obtained by heating SiO_2_ glass, was used as a starting material. After laser heating the cristobalite and CO_2_ mixture at conditions similar to those used in the first experiment, we could identify Si[CO_3_]_2_ by its distinct Raman spectroscopic signature (fig. S7). Moreover, the anhydrous composition is charge balanced. Also, our DFT calculations confirm the anhydrous structural model. Furthermore, no Raman signal at high wave numbers (3000 to 3500 cm^−1^) has been observed after heating the cristobalite + CO_2_ mixture, which could be indicative for the presence of O─H bonds.

We used our DFT calculations to obtain the *p,V* relation for Si[CO_3_]_2_-*P*2_1_/*n* between 0 and 50 GPa. The calculated *p*,*V* data were used to derive the bulk modulus (*K*_0_) and its pressure derivative (*K*_p_). From an EoS fit to the *p*,*V* data (fig. S4), we obtained a bulk modulus of *K*_0_ = 53.1(4) GPa with *K*_p_ = 6.40(5). The ambient pressure bulk modulus is in good agreement with the one derived from our stress-strain calculations [52(1) GPa]. Without using a correction scheme for van der Waals (vdW) interactions during the calculations, the bulk modulus derived from the EoS fit is noticeably lower [*K*_0_ = 37(1) GPa with *K*_p_ = 7.3(1)]. The elastic stiffness coefficients are listed in table S3. The bulk modulus is noticeably lower than for other *sp*^2^ carbonates hosting sixfold coordinated cations, such as Ca[CO_3_] [67(2) GPa], Mg[CO_3_] [107(1) GPa], or Be[CO_3_] [153.0(3) GPa] (*32*, *42*). In contrast, the pressure derivative of the bulk modulus is noticeably higher for Si[CO_3_]_2_-*P*2_1_/*n*. The compression mechanism for Si[CO_3_]_2_-*P*2_1_/*n* is strongly anisotropic. Si[CO_3_]_2_-*P*2_1_/*n* is highly compressible along the *b* axis and incompressible along the *a* and *c* axes (fig. S5). This compression behavior can be explained by the layered crystal structure of Si[CO_3_]_2_-*P*2_1_/*n*. The [CO_3_]^2−^ groups within the *a*, *c* planes are incompressible, but during compression, the space between the [CO_3_]^2−^ group layers is strongly reduced. Experimentally, the monoclinic angle is always found to be very close to 90° at all pressures. Because of the limited access to the reciprocal space in the DAC, the error on the lattice parameter may be too large to distinguish between orthorhombic and monoclinic symmetry. However, the crystal structure could not be solved in a higher space group symmetry. This is consistent with our DFT-based calculations, which show that the relaxed structures always maintain the monoclinic *P*2_1_/*n* space group symmetry over the whole investigated pressure range while the monoclinic angle remains between 90.19° (50 GPa) and 90.35° (0 GPa).

## DISCUSSION

We have synthesized the anhydrous silicon *sp*^2^ carbonate Si[CO_3_]_2_-*P*2_1_/*n* through the reaction of silicic acid and CO_2_ and by the reaction of cristobalite with CO_2_ under lower-mantle pressure-temperature conditions [40(2) GPa and 1800(200) K]. At ≈40 GPa, the density of Si[CO_3_]_2_-*P*2_1_/*n* is approximately 3.9 g cm^−3^, slightly lower than that of typical uppermost lower-mantle assemblages ($\approx 4.4\;\text{to}\;4.6\;g\;{\text{cm}}^{-3}$) (*23*). Thus, once formed, this phase would have neutral to slightly negative buoyancy and could represent a localized, but viable, host for carbon in the deep mantle. At 40 GPa, the seismic velocities computed from the elastic stiffness tensor are *v*_p_ = 11.3 km s^−1^ and *v*_s_ = 6.3 km s^−1^, which is very close to the corresponding values of the preliminary reference earth model (PREM) (*23*), so that the presence of Si[CO_3_]_2_ would not have an identifiable seismic signature. To understand whether Si[CO_3_]_2_-*P*2_1_/*n* may occur at the *p,T,x* conditions of Earth’s lower mantle, it is now of relevance to benchmark its stability in the presence of the main mantle minerals.

## MATERIALS AND METHODS

A detailed description of the experimental and computational methods is available in the Supplementary Materials.

### Sample synthesis

The high-pressure experiments were carried out using laser-heated Boehler-Almax–type DACs (*43*). For the loading of the DAC, we used in the first set of experiments commercial silicic acid [SiO*_x_*(OH)_4–2*x*_] powder (99.9% purity, Sigma-Aldrich, Merck KGaA, Darmstadt, Germany) without further purification. In the second set of experiments, we used phase pure synthetic cristobalite (SiO_2_) obtained by heating silica glass to 1823 K in an oxygen-controlled high-temperature oven for 6 hours following an earlier approach (*44*). CO_2_ gas was used as purchased (Nippon gases, purity ≥99.996%) and loaded as dry ice by cryogenic loading into the DACs using a custom-built cryogenic loading system (*45*). The DAC was cooled down to ≈120 K for the cryogenic loading. CO_2_-I (dry ice) was directly condensed from a gas jet into the sample chamber. The pressure during compression was determined from the position of the high-frequency edge of the diamond Raman band (*46*). At the target pressure of the experiment, laser heating was performed from both sides using a custom-built setup equipped with a Coherent Diamond K-250 pulsed CO_2_ laser (λ = 10,600 nm) (*6*).

### Sample characterization

Raman spectroscopy was performed in DACs at elevated pressures using an Oxford Instruments WITec alpha 300R Raman imaging microscope. The WITec alpha 300R was equipped with an Olympus SLMPan N 50× objective, and the measurements were performed using a 532-nm laser and the 1800 grooves mm^−1^ grating of the WITec UHTS 300S (VIS-NIR) spectrograph. Raman maps were measured on a grid with a step size of 0.5 μm. Single-crystal synchrotron XRD was carried out at two different synchrotron beamlines. At 40(2) GPa, the experiments were carried out at the synchrotron PETRA III (DESY) in Hamburg, Germany, at the Extreme Conditions Beamline P02.2 (*47*). The beam size on the sample was $\approx 2\times 2\;{\mathrm{\mu m}}^{2}$ [full width at half maximum (FWHM)] using a wavelength of 0.2903 Å (43.7 keV), and the diffraction data were collected using a Perkin Elmer XRD1621 detector. At 6(1) GPa and ambient conditions, the experiments were carried out at the European Synchrotron Radiation Facility (ESRF) in Grenoble, France, at the Materials Science Beamline line ID11 (*48*). The beam size on the sample was $\approx 0.6\times 0.6\;{\mathrm{\mu m}}^{2}$ (FWHM) using a wavelength of 0.2846 Å (43.6 keV), and the diffraction data were collected using an Eiger2 X 4M CdTe detector. We rotated the DAC by ±34° around the vertical axis perpendicular to the beam while collecting frames in 0.25° steps with 2 s acquisition time per frame. The structure solution and refinement on the single-crystal diffraction data were performed using the software package OLEX2 using SHELXT for the crystal structure determination and SHELXL for the refinement (*49*–*51*).

### DFT-based calculations

First-principles calculations were carried out within the framework of DFT, using the Perdew-Burke-Ernzerhof (PBE) exchange-correlation functional and the plane wave/pseudopotential approach implemented in the CASTEP simulation package (*52*–*54*). CASTEP and auxiliary programs, including those for a symmetry analysis, an analysis of the electron density distribution, and for visualizations, were run within the BIOVIA Materials Studio suite of programs (*55*). We used the correction scheme for vdW interactions developed by Tkatchenko and Scheffler (*56*). Phonon frequencies were obtained from density functional perturbation theory (DFPT) calculations (*57*, *58*). Raman intensities were computed using DFPT with the “2*n* + 1” theorem approach (*59*).

## Supplementary Materials

### This PDF file includes:

Supplementary Materials and Methods Supplementary Text Figs. S1 to S7 Tables S1 to S3 References

## References

[1] R. J. Reeder, *Carbonates: Mineralogy and Chemistry* (De Gruyter, 1983).

[2] N. R. McKenzie, B. K. Horton, S. E. Loomis, D. F. Stockli, N. J. Planavsky, C.-T. A. Lee, Continental arc volcanism as the principal driver of icehouse-greenhouse variability. Science 352, 444–447 (2016). 27102480 10.1126/science.aad5787

[3] M. M. Hirschmann, Comparative deep Earth volatile cycles: The case for C recycling from exosphere/mantle fractionation of major (H_2_O, C, N) volatiles and from H_2_O/Ce, CO_2_/Ba, and CO_2_/Nb exosphere ratios. Earth Planet. Sci. Lett. 502, 262–273 (2018).

[4] P. B. Kelemen, C. E. Manning, Reevaluating carbon fluxes in subduction zones, what goes down, mostly comes up. Proc. Natl. Acad. Sci. U.S.A. 11, E3997–E4006 (2015). 10.1073/pnas.1507889112 PMC4522802 26048906

[5] P. D. Clift, A revised budget for Cenozoic sedimentary carbon subduction. Rev. Geophys. 55, 97–125 (2017).

[6] L. Bayarjargal, C.-J. Fruhner, N. Schrodt, B. Winkler, CaCO_3_ phase diagram studied with Raman spectroscopy at pressures up to 50 GPa and high temperatures and DFT modeling. Phys. Earth Planet. Inter. 281, 31–45 (2018).

[7] J. Binck, S. Chariton, M. Stekiel, L. Bayarjargal, W. Morgenroth, V. Milman, L. Dubrovinsky, B. Winkler, High-pressure, high-temperature phase stability of iron-poor dolomite and the structures of dolomite-IIIc and dolomite-V. Phys. Earth Planet. Inter. 299, 106403 (2020).

[8] S. Kakizawa, T. Inoue, H. Suenami, T. Kikegawa, Decarbonation and melting in MgCO_3_–SiO_2_ system at high temperature and high pressure. J. Mineral. Petrol. Sci. 110, 179–188 (2015).

[9] X. Li, Z. Zhang, J.-F. Lin, H. Ni, V. B. Prakapenka, Z. Mao, New high-pressure phase of CaCO_3_ at the topmost lower mantle: Implication for the deep-mantle carbon transportation. Geophys. Res. Lett. 45, 1355–1360 (2018).

[10] J. Gao, X. Wu, X. Yuan, W. Su, Fate of carbonates in the Earth’s mantle (10-136 GPa). Front. Earth Sci. 10, 837775 (2018).

[11] D. Spahr, J. König, L. Bayarjargal, V. Milman, A. Perlov, H.-P. Liermann, B. Winkler, Sr[C_2_O_5_] is an inorganic pyrocarbonate salt with [C_2_O_5_]^2−^ complex anions. J. Am. Chem. Soc. 144, 2899–2904 (2022). 35134291 10.1021/jacs.2c00351

[12] D. J. Spahr, L. Bayarjargal, M. Bykov, L. Brüning, T. H. Reuter, V. Milman, H.-P. Liermann, B. Winkler, High-pressure synthesis of acentric sodium pyrocarbonate, Na_2_[C_2_O_5_]. Dalton Trans. 53, 40–44 (2023). 38054559 10.1039/d3dt03673a

[13] D. Spahr, L. Bayarjargal, M. Bykov, L. Brüning, P. L. Jurzick, Y. Wang, V. Milman, K. Refson, M. Mezouar, B. Winkler, Ca_3_[C_2_O_5_]_2_[CO_3_] is a pyrocarbonate which can be formed at conditions prevalent in the Earth’s transition zone. Commun. Chem. 7, 238 (2024). 39433974 10.1038/s42004-024-01293-1 PMC11494096

[14] M. Santoro, F. Gorelli, J. Haines, O. Cambon, C. Levelut, G. Garbarino, Silicon carbonate phase formed from carbon dioxide and silica under pressure. Proc. Natl. Acad. Sci. U.S.A. 108, 7689–7692 (2011). 21518903 10.1073/pnas.1019691108 PMC3093504

[15] M. Santoro, F. A. Gorelli, R. Bini, A. Salamat, G. Garbarino, C. Levelut, O. Cambon, J. Haines, Carbon enters silica forming a cristobalite-type CO_2_–SiO_2_ solid solution. Nat. Commun. 5, 3761 (2014). 24781844 10.1038/ncomms4761 PMC5603768

[16] D. Santamaria-Perez, C. McGuire, A. Makhluf, A. Kavner, R. Chuliá-Jordan, J. L. Jorda, F. Rey, J. Pellicer-Porres, D. Martinez-Garcá, P. Rodriguez-Hernández, A. Muñoz, Strongly-driven Re + CO_2_ redox reaction at high-pressure and high-temperature. Nat. Commun. 7, 13647 (2016). 27897171 10.1038/ncomms13647 PMC5141295

[17] D. Santamaria-Perez, T. Marqueño, S. MacLeod, J. Ruiz-Fuertes, D. Daisenberger, R. Chuliá-Jordan, D. Errandonea, J. Luis Jordá, F. Rey, C. McGuire, A. Makhluf, A. Kavner, C. Popescu, Structural evolution of CO_2_-filled pure silica LTA zeolite under high-pressure high-temperature conditions. Chem. Mater. 29, 4502–4510 (2017).

[18] A. Morales-García, M. Marqués, J. M. Menéndez, D. Santamaría-Pérez, V. G. Baonza, J. M. Recio, First-principles study of structure and stability in Si-C-O-based materials. Theor. Chem. Acc. 132, 1308 (2013).

[19] R. Zhou, B. Qu, J. Dai, X. C. Zeng, Unraveling crystalline structure of high-pressure phase of silicon carbonate. Phys. Rev. X 4, 011030 (2014).

[20] M. Marqués, A. Morales-García, J. M. Menéndez, V. G. Baonza, J. M. Recio, A novel crystalline SiCO compound. Phys. Chem. Chem. Phys. 17, 25055–25060 (2015). 26345349 10.1039/c5cp03673a

[21] B. Qu, D. Li, L. Wang, J. Wu, R. Zhou, B. Zhang, X. C. Zeng, Mechanistic study of pressure and temperature dependent structural changes in reactive formation of silicon carbonate. RSC Adv. 6, 26650–26657 (2016).

[22] X. Yong, J. S. Tse, J. Chen, Mechanism of chemical reactions between SiO_2_ and CO_2_ under mantle conditions. ACS Earth Space Chem. 2, 548–555 (2018).

[23] A. M. Dziewonski, D. L. Anderson, Preliminary reference Earth model. Phys. Earth Planet. Inter. 25, 297–356 (1981).

[24] J. Ritsema, W. Xu, L. Stixrude, C. Lithgow-Bertelloni, Estimates of the transition zone temperature in a mechanically mixed upper mantle. EPSL 277, 244–252 (2009).

[25] T. Katsura, A. Yoneda, D. Yamazaki, T. Yoshino, E. Ito, Adiabatic temperature profile in the mantle. Phys. Earth Planet. Inter. 183, 212–218 (2010).

[26] P. Nimis, M. Alvaro, F. Nestola, R. J. Angel, K. Marquardt, G. Rustioni, J. W. Harris, F. Marone, First evidence of hydrous silicic fluid films around solid inclusions in gem-quality diamonds. Lithos 260, 384–389 (2016).

[27] D. Scelta, K. F. Dziubek, M. Ende, R. Miletich, M. Mezouar, G. Garbarino, R. Bini, Extending the stability field of polymeric carbon dioxide phase V beyond the Earth’s geotherm. Phys. Rev. Lett. 126, 065701 (2021). 33635684 10.1103/PhysRevLett.126.065701

[28] F. Datchi, B. Mallick, A. Salamat, S. Ninet, Structure of polymeric carbon dioxide CO_2_-V. Phys. Rev. Lett. 108, 125701 (2012). 22540597 10.1103/PhysRevLett.108.125701

[29] K. Aoki, H. Yamawaki, M. Sakashita, Y. Gotoh, K. Takemura, Crystal structure of the high-pressure phase of solid CO_2_. Science 263, 356–358 (1994). 17769798 10.1126/science.263.5145.356

[30] H. Olijnyk, A. P. Jephcoat, Vibrational studies on CO_2_ up to 40 GPa by Raman spectroscopy at room temperature. Phys. Rev. B 57, 879–888 (1998).

[31] J. Binck, L. Bayarjargal, S. S. Lobanov, W. Morgenroth, R. Luchitskaia, C. J. Pickard, V. Milman, K. Refson, D. B. Jochym, P. Byrne, B. Winkler, Phase stabilities of MgCO_3_ and MgCO_3_-II studied by Raman spectroscopy, X-ray diffraction, and density functional theory calculations. Phys. Rev. Mater. 4, 055001 (2020).

[32] D. Spahr, L. Bayarjargal, E. Bykova, M. Bykov, L. Brüning, V. Kovalev, V. Milman, J. Wright, B. Winkler, 6-Fold-coordinated beryllium in calcite-type be Be[CO_3_]. Inorg. Chem. 63, 19513–19517 (2024). 39383049 10.1021/acs.inorgchem.4c03681

[33] N. Biedermann, S. Speziale, B. Winkler, H. J. Reichmann, M. Koch-Müller, G. Heide, High-pressure phase behavior of SrCO_3_: An experimental and computational Raman scattering study. Phys. Chem. Minerals 44, 335–343 (2017).

[34] D. Spahr, L. Bayarjargal, E. Bykova, M. Bykov, T. H. Reuter, L. Brüning, P. L. Jurzick, L. Wedek, V. Milman, B. Wehinger, B. Winkler, Synthesis and crystal structure of acentric anhydrous beryllium carbonate Be(CO_3_). Chem. Commun. 60, 10208–10211 (2024). 10.1039/d4cc03462g 39206736

[35] G. Fiquet, F. Guyot, M. Kunz, J. Matas, D. Andrault, M. Hanfland, Structural refinements of magnesite at very high pressure. Am. Mineral. 87, 1261–1265 (2002).

[36] Y. Zhang, S. Chariton, J. He, S. Fu, F. Xu, V. B. Prakapenka, J.-F. Lin, Atomistic insight into the ferroelastic post-stishovite transition by high-pressure single-crystal X-ray diffraction. Am. Mineral. 108, 110–119 (2023).

[37] H. Effenberger, K. Mereiter, J. Zemann, Crystal structure refinements of magnesite, calcite, rhodochrosite, siderite, smithonite, and dolomite, with discussion of some aspects of the stereochemistry of calcite type carbonates. Z. Kristallogr. 156, 233–243 (1981).

[38] L. Bayarjargal, D. Spahr, V. Milman, J. Marquardt, N. Giordano, B. Winkler, Anhydrous aluminium carbonates and isostructural compounds. Inorg. Chem. 62, 13910–13918 (2023). 37579301 10.1021/acs.inorgchem.3c01832

[39] R. D. Shannon, Revised effective ionic radii and systematic studies of interatomic distances in halides and chalcogenides. Acta Crystallogr. 32, 751–767 (1976).

[40] A. H. Davis, N. V. Solomatova, R. Caracas, A. J. Campbell, Carbon storage in Earth’s deep interior implied by carbonate-silicate-iron melt miscibility. Geochem. Geophys. Geosyst. 24, e2023GC010896 (2023).

[41] D. Spahr, L. Bayarjargal, E. Bykova, M. Bykov, G. L. Murphy, P. Kegler, V. Milman, N. Giordano, B. Winkler, High-pressure synthesis of U_2_[CO_3_]_3_ and U[CO_3_]_2_ as potential host phases for uranium in the Earth’s mantle. Commun. Chem. 9, 112 (2026). 41617977 10.1038/s42004-026-01911-0 PMC12960825

[42] J. Zhang, R. J. Reeder, Comparative compressibilities of calcite-structure carbonates: Deviations from empirical relations. Am. Mineral. 84, 861–870 (1999).

[43] R. Boehler, New diamond cell for single–crystal X-ray diffraction. Rev. Sci. Instrum. 77, 115103 (2006).

[44] X. Li, X. Yin, L. Zhang, S. He, The devitrification kinetics of silica powder heat-treated in different conditions. J. Non Cryst. Solids 354, 3254–3259 (2008).

[45] D. Spahr, L. Bayarjargal, L. Brüning, V. Kovalev, E. Bykova, M. Bykov, V. Milman, M. Mezouar, B. Winkler, Synthesis and crystal structure of anhydrous di-iodyl carbonate (IO_2_)_2_[CO_3_], hosting I^5+^-cations. JACS Au 5, 4675–4680 (2025). 41169570 10.1021/jacsau.5c00829 PMC12569702

[46] Y. Akahama, H. Kawamura, Pressure calibration of diamond anvil Raman gauge to 310 GPa. J. Appl. Phys. 100, 043516 (2006).

[47] H.-P. Liermann, Z. Konôpková, W. Morgenroth, K. Glazyrin, J. Bednarčik, E. E. McBride, S. Petitgirard, J. T. Delitz, M. Wendt, Y. Bican, A. Ehnes, I. Schwark, A. Rothkirch, M. Tischer, J. Heuer, H. Schulte-Schrepping, T. Kracht, H. Franz, The extreme conditions beamline P02.2 and the extreme conditions science infrastructure at PETRAIII. J. Synchrotron Radiat. 22, 908–924 (2015). 26134794 10.1107/S1600577515005937 PMC4489534

[48] J. Wright, C. Giacobbe, M. Majku, New opportunities at the materials science beamline at ESRF to exploit high energy nano-focus X-ray beams. Curr. Opin. Solid. St. M. 24, 100818 (2020).

[49] O. V. Dolomanov, L. J. Bourhis, R. J. Gildea, J. A. K. Howard, H. Puschmann, *OLEX2*: A complete structure solution, refinement and analysis program. J. Appl. Cryst. 42, 339–341 (2009).

[50] G. M. Sheldrick, *SHELXT*—Integrated space-group and crystal-structure determination. Acta Crystallogr. 71, 3–8 (2015). 10.1107/S2053273314026370 PMC4283466 25537383

[51] G. M. Sheldrick, Crystal structure refinement with *SHELXL*. Acta Crystallogr. 71, 3–8 (2015). 10.1107/S2053229614024218 PMC4294323 25567568

[52] P. Hohenberg, W. Kohn, Inhomogeneous electron gas. Phys. Rev. 136, B864–B871 (1964).

[53] J. P. Perdew, K. Burke, M. Ernzerhof, Generalized gradient approximation made simple. Phys. Rev. Lett. 77, 3865–3868 (1996). 10062328 10.1103/PhysRevLett.77.3865

[54] S. J. Clark, M. D. Segall, C. J. Pickard, P. J. Hasnip, M. I. J. Probert, K. Refson, M. C. Payne, First principles methods using CASTEP. Z. Kristallogr. 220, 567–570 (2005).

[55] BIOVIA, Materials Studio, San Diego, USA (2025).

[56] A. Tkatchenko, M. Scheffler, Accurate molecular van der Waals interactions from ground-state electron density and free-atom reference data. Phys. Rev. Lett. 102, 073005 (2009). 19257665 10.1103/PhysRevLett.102.073005

[57] S. Baroni, S. de Gironcoli, A. Dal Corso, P. Giannozzi, Phonons and related crystal properties from density-functional perturbation theory. Rev. Mod. Phys. 73, 515–562 (2001).

[58] K. Refson, P. R. Tulip, S. J. Clark, Variational density-functional perturbation theory for dielectrics and lattice dynamics. Phys. Rev. B 73, 155114 (2006).

[59] K. Miwa, Prediction of Raman spectra with ultrasoft pseudopotentials. Phys. Rev. B 84, 094304 (2011).

[60] C. S. Yoo, H. Cynn, F. Gygi, G. Galli, V. Iota, M. Nicol, S. Carlson, D. Häusermann, C. Mailhiot, Crystal structure of carbon dioxide at high pressure: “Superhard” polymeric carbon dioxide. Phys. Rev. Lett. 83, 5527–5530 (1999).

[61] L. R. Benedetti, P. Loubeyre, Temperature gradients, wavelength-dependent emissivity, and accuracy of high and very-high temperatures measured in the laser-heated diamond cell. High Press. Res. 24, 423–445 (2004).

[62] Z. Du, G. Amulele, L. R. Benedetti, K. K. M. Lee, Mapping temperatures and temperature gradients during flash heating in a diamond-anvil cell. Rev. Sci. Instrum. 84, 075111 (2013). 23902110 10.1063/1.4813704

[63] M. Wojdyr, *Fityk*: A general-purpose peak fitting program. J. Appl. Cryst. 43, 1126–1128 (2010).

[64] C. Prescher, V. B. Prakapenka, *DIOPTAS*: A program for reduction of two-dimensional X-ray diffraction data and data exploration. High. Press. Res. 35, 223–230 (2015).

[65] Agilent, CrysAlis PRO, Yarnton, England (2014).

[66] A. Aslandukov, M. Aslandukov, N. Dubrovinskaia, L. Dubrovinsky, *Domain Auto Finder* (*DAFi*) program: The analysis of single-crystal X-ray diffraction data from polycrystalline samples. J. Appl. Cryst. 55, 1383–1391 (2022). 36249501 10.1107/S1600576722008081 PMC9533752

[67] K. Lejaeghere, G. Bihlmayer, T. Björkman, P. Blaha, S. Blügel, V. Blum, D. Caliste, I. E. Castelli, S. J. Clark, A. D. Corso, S. de Gironcoli, T. Deutsch, J. K. Dewhurst, I. Di Marco, C. Draxl, M. Dułak, O. Eriksson, J. A. Flores-Livas, K. F. Garrity, L. Genovese, P. Giannozzi, M. Giantomassi, S. Goedecker, X. Gonze, O. Granas, E. K. U. Gross, A. Gulans, F. Gygi, D. R. Hamann, P. J. Hasnip, N. A. W. Holzwarth, D. Iusan, D. B. Jochym, F. Jollet, D. Jones, G. Kresse, K. Koepernik, E. Kücükbenli, Y. O. Kvashnin, I. L. M. Locht, S. Lubeck, M. Marsman, N. Marzari, U. Nitzsche, L. Nordström, T. Ozaki, L. Paulatto, C. J. Pickard, W. Poelmans, M. I. J. Probert, K. Refson, M. Richter, G.-M. Rignanese, S. Saha, M. Scheffler, M. Schlipf, K. Schwarz, S. Sharma, F. Tavazza, P. Thunström, A. Tkatchenko, M. Torrent, D. Vanderbilt, M. J. van Setten, V. Van Speybroeck, J. M. Wills, J. R. Yates, G.-O. Zhang, S. Cottenier, Reproducibility in density functional theory calculations of solids. Science 351, aad3000 (2016). 27013736 10.1126/science.aad3000

[68] H. J. Monkhorst, J. D. Pack, Special points for Brillouin-zone integrations. Phys. Rev. B 13, 5188–5192 (1976).

[69] P. Vinet, J. H. Rose, J. Ferrante, J. R. Smith, Universal features of the equation of state of solids. Phys. Condens. Matter 1, 1941–1963 (1989). 10.1103/physrevb.35.1945 9941621

[70] J. Gonzalez-Platas, M. Alvaro, F. Nestola, R. Angel, *EosFit7-GUI*: A new graphical user interface for equation of state calculations, analyses and teaching. J. Appl. Cryst. 49, 1377–1382 (2016).

