# Supplementary material for: Silicon carbonate, Si[CO3]2, is a potential carbon host in the deep Earth
Source: Sci Adv. 2026 May 29;12(22):eaee5766. doi: 10.1126/sciadv.aee5766 (PMC13220855; doi:10.1126/sciadv.aee5766)
Supplement: Supplementary file 1 — Supplementary Materials and Methods Supplementary Text Figs. S1 to S7 Tables S1 to S3 References [file sciadv.aee5766_sm.pdf]

Science Advances
AAAS

Supplementary Materials for

Silicon carbonate, $Si[CO_3]_2$, is a potential carbon host in the deep Earth

Dominik Spahr *et al.*

Corresponding author: Dominik Spahr, d.spahr@kristall.uni-frankfurt.de

Sci. Adv. **12**, eaee5766 (2026)
DOI: 10.1126/sciadv.aee5766

This PDF file includes:

Supplementary Materials and Methods
Supplementary Text
Figs. S1 to S7
Tables S1 to S3
References

Supplementary Materials & Methods

Sample material

In the first set of experiments we used commercial silicic acid ($SiO_x(OH)_{4-2x}$) powder (99.9% purity, Sigma-Aldrich, Merck KGaA, Darmstadt, Germany) as starting material for the high-pressure experiments. The silicic acid powder was used as received without further purification. In the second set of experiments we used phase pure synthetic cristobalite (SiO_2) as starting material. The cristobalite was obtained by heating silica glass to 1823 K in an oxygen-controlled high-temperature oven for 6 hours following an earlier approach (*44*). The sample powders were compacted between a diamond and a glass plate. Afterwards, a $\approx 40 \times 40 \times 10$ µm^3-sized powder compact was selected for the loading of the of the diamond anvil cell (DAC). The CO_2 gas for the gas-jet was used as purchased (Nippon gases, purity ≥ 99.996%).

High-pressure experiments

The high-pressure experiments were carried out in Boehler-Almax type DACs equipped with diamonds having an opening angle of 70° and 300 µm sized culets on both sides (*43*). We used Re-gaskets, which were pre-indented to thicknesses of ≈ 40 µm. Afterwards, sample chambers with ≈ 120 µm diameter were drilled into the Re-gaskets using a custom-built laser set-up. The pressure during compression of the DAC was derived from the position of the high frequency edge of the diamond Raman band and we assume an error of at least 5% due to non-hydrostatic conditions in the DAC (*46*). In addition, we expect that the pressure conditions in the DAC before laser heating are very likely non-hydrostatic as CO_2-III may sustain pressure gradients up to 0.2 GPa µm^{-1} at high pressures without heating (*60*).

In the first step, the $\approx 40 \times 40 \times 10$ µm^3-sized silicic acid or cristobalite powder compact was placed on the culet of the bottom diamond of the DAC. In a second step, the DAC was placed on a liquid nitrogen cooled Cu-holder and cooled down for the cryogenic loading using a custom-built cryogenic loading system (see Spahr *et al.* (*45*)). The DAC was cooled down to ≈ 120 K and CO_2-I (dry ice) was directly condensed into the sample chamber from the CO_2 gas jet. We used a small nozzle to align the CO_2 gas jet with 5 l min^{-1} directly on the gap between upper diamond and the

gasket. We used argon (5 l min^{-1}) as a purge gas. During the loading process the precipitation of the CO_2 in the sample chamber was monitored using an optical microscope and equipped with a camera. After the sample chamber was completely covered with dry-ice, the enclosure was opened and the DAC was tightly closed. Finally, the sample in the DAC was compressed to the target pressure of the experiment without intermediate heating.

Laser heating

The silicic acid or cristobalite in the CO_2 environment was laser-heated from both sides at the target pressure of the experiment (40(2) GPa) using a custom-built set-up equipped with a Coherent Diamond K-250 pulsed CO_2 laser (λ = 10600 nm) (*6*). The laser power was adjusted to achieve a coupling of the laser to the sample, using a laser power between 1–6 W. The maximum temperature achieved during the laser-heating was T_{max} = 1800(200) K. The temperatures were determined by the two-color pyrometer method, employing Planck and Wien fits (*61*). The heating time during the experiments was ≈ 60 min. It is well established that laser-heating in DACs always suffers from large temperature gradients and the actual temperature is strongly dependent on the coupling of the laser with the sample, especially at lower temperatures. At these low temperatures we estimate an uncertainty of at least ±15% of the nominal temperature in the laser-heated region depending on the focus of the laser beam, based on typical 2D temperature-gradient determination experiments performed in DACs (*62*).

Raman spectroscopy

Raman spectroscopy was performed in the DACs using an Oxford Instruments WITec alpha 300R Raman imaging microscope. The Raman microscope was equipped with an Olympus SLMPan N 50× objective. The measurements were performed using the 532 nm laser. We employed the 1800 grooves mm^{-1} grating of the WITec UHTS 300S (VIS-NIR) spectrograph in combination with an Andor DR316B-LDC-DD CCD detector for the measurements. The laser power was 100 mW on the sample and the spot size of the Raman laser was ≈ 0.8 µm. We assume a depth resolution of ≈ 6 µm in the direction of the laser beam. Raman maps were measured on a grid with a step-size of 0.5 µm. The background of the Raman spectra was corrected using the software package Fityk (*63*).

Single-crystal synchrotron X-ray diffraction

Single-crystal synchrotron X-ray diffraction at 40(2) GPa was carried out at the synchrotron PETRA III (DESY) in Hamburg, Germany, at the Extreme Conditions Beamline P02.2 (*47*). The beam size on the sample was $\approx 2\times2$ µm^2 (FWHM), focused by Kirkpatrick Baez mirrors. The diffraction data were collected using a Perkin Elmer XRD1621 detector, a wavelength of 0.2903 Å (42.7 keV) and a sample to detector distance of 400.4 mm. Single-crystal diffraction at 6(1) GPa as well as at ambient conditions was carried out at the ESRF in Grenoble, France, at the Materials Science Beamline line ID11 (*48*). The beam size on the sample was $\approx 0.6\times0.6$ µm^2 (FWHM). The diffraction data were collected using an Eiger2 X 4M CdTe detector, a wavelength of 0.2846 Å (43.6 keV) and a detector to sample distance of 183 mm. We rotated the DAC by ±33° around the vertical axis perpendicular to the beam while collecting frames in 0.25° steps with 2 s acquisition time per frame.

The sample to detector distance was calibrated using the powder diffraction pattern of a CeO_2 standard in conjunction with the software DIOPTAS (*64*). The diffractometer/detector geometry for the analysis of the single crystal diffraction data was calibrated using diffraction data collected from a single crystal of enstatite ($MgSiO_3$) in a DAC at ambient pressure at P02.2 and using a siliconsingle-crystal at ambient pressure at ID11. After the data collection, the reflections were indexed and integrated employing CrysAlis^{PRO} (version 43.67a) (*65*). We used the Domain Auto Finder program (DAFi) to find possible single-crystal domains for the subsequent data reduction (*66*). The structure solution and refinement were performed using the software package OLEX2 employing SHELXT for the crystal structure determination and SHELXL for the refinement (*49–51*).

Density functional theory-based calculations

First-principles calculations were carried out within the framework of density functional theory (DFT), employing the Perdew-Burke-Ernzerhof (PBE) exchange-correlation functional and the plane wave/pseudopotential approach implemented in the CASTEP simulation package (*52–54*). CASTEP and auxiliary programs, including those for a symmetry analysis, an analysis of the electron density distribution, and for visualizations, were run within the BIOVIA Materials Studio suite of programs (*55*).

"On the fly" norm-conserving or ultrasoft pseudopotentials generated using the descriptors in the CASTEP data base were employed in conjunction with plane waves up to a kinetic energy cutoff of 1020 eV or 630 eV, for norm-conserving and ultrasoft pseudopotentials, respectively. The accuracy of the pseudopotentials is well established (*67*). A correction scheme for van der Waals (v.d.W.) interactions was applied in the DFT-calculations. We employed the correction scheme developed by Tkatchenko and Scheffler (*56*). A Monkhorst-Pack grid was used for Brillouin zone integrations (*68*). We used a distance between grid points of <0.023 Å^{-1}. Convergence criteria for geometry optimization included an energy change of $<5 \times 10^{-6}$ eV atom^{-1} between steps, a maximal force of <0.008 eV Å^{-1} and a maximal component of the stress tensor <0.02 GPa. Phonon frequencies were obtained from density functional perturbation theory (DFPT) calculations (*57,58*). Raman intensities were computed using DFPT with the "$2n + 1$" theorem approach (*59*).

Supplementary Text

Single-crystal synchrotron X-ray diffraction

First, we collected X-ray diffraction data at 40(2) GPa on a 2D grid across the sample chamber using a spot size of $\approx 2 \times 2$ µm^2 in order to locate promising positions for the collection of single-crystal diffraction data. Afterwards, we performed synchrotron single-crystal X-ray diffraction on selected locations of the grid where unidentified reflections were present. Fig. S1 a shows a part of the reciprocal space reconstruction for the $(\bar{1}kl)$ plane of the single-crystal on which the crystal structure was determined, demonstrating the high quality of the collected diffraction data at 40(2) GPa. In addition to the reflection of the unknown phase, we observed reflections and powder rings of CO_2-V and of the diamonds in the diffraction data. Fig. S1 b shows the projection of the reciprocal space along a^* (top) and b^* (bottom). The effect of the shading of diffracted beams due to the metallic body of the DAC can be observed.

From the single crystal diffraction data collected at 40(2) GPa, we solved the crystal structure of the unknown phase in the monoclinic space group $P2_1/n$ (No. 14) with $Z = 4$ and $Si[CO_3]_2$ composition (Table. S1). The low R_1-value of (4.1%) in combination with a reasonable reflection to parameter ratio is acceptable (7.5:1) reveals a convincing structure refinement. The displacement parameters of all atoms (silicon, carbon and oxygen) were refined anisotropically. No constraints or restraints were applied for the refinement. The experimental crystallographic parameters at 40(2) GPa are in good agreement with the data derived from the DFT calculations: a = 4.1824 Å, b = 4.1679 Å, c = 7.2291 Å and β = 90.19° (V = 126.02 Å^3).

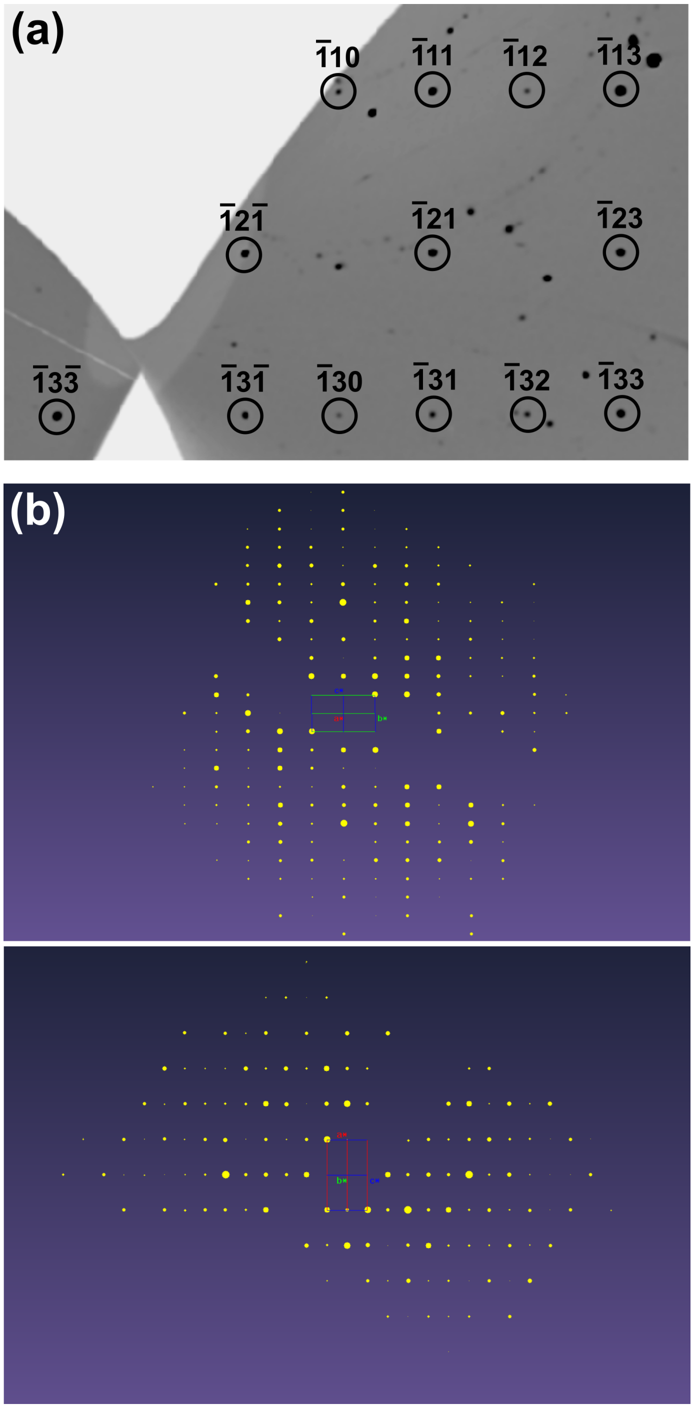

Figure S1: (a) Reciprocal space reconstruction for the $(\bar{1}kl)$ plane at 40(2) GPa. (b) Schematic depiction of the reflections in reciprocal space using the Ewald-Explorer in CrysAlis after data reduction which were later used for the refinement. Projections of the reciprocal space are shown along a^* (top) and b^* (bottom).

Table S1: Structural parameters of $Si[CO_3]_2$-$P2_1/n$ at 40(2) GPa (P02.2, PETRA III) and at 6(1) GPa (ID11, ESRF) from single-crystal structure solution (ambient temperature).

	**P02.2 (PETRA III) at 40(2) GPa**	**ID11 (ESRF) at 6(1) GPa**
**Crystal data**		
Crystal system	monoclinic	monoclinic
Space group	$P2_1/n$	$P2_1/n$
Chemical formula	$Si[CO_3]_2$	$Si[CO_3]_2$
M_r (g mol^{-1})	147.97	150.92
a (Å)	4.1412(19)	4.335(3)
b (Å)	4.1916(9)	4.6573(8)
c (Å)	7.2283(7)	7.4205(11)
α (°)	90.0	90.0
β (°)	89.88(2)	90.25(3)
γ (°)	90.0	90.0
V (Å^3)	125.47(6)	149.8(1)
Z	2	2
**Data collection**		-
F_{000}	148	151
θ range (°)	2.31–17.47	2.07–15.50
measured reflections	650	516
independent reflections	376	304
reflections $I > 2\sigma(I)$	286	244
R_{int}	0.014	0.020
		-
**Refinement**		-
$R_1[I > 2\sigma(I)]$, $wR_2(I)$	0.041, 0.102	0.053, 0.123
No. of reflections	376	304
No. of parameters	50	45
No. of restraints	0	-
No. of constraints	0	1
$\Delta\rho_{max}$, $\Delta\rho_{min}$ (e Å^{-3})	0.53, −0.45	0.52, −0.50

After decompression of the sample to 6(1) GPa we refined the crystal structure of Si[CO_3]$_2$-$P2_1/n$ from the diffraction data collected at beamline ID11 at the ESRF (Table. S1). The low R_1-value of (5.3%) in combination with a acceptable reflection to parameter (6.8:1) reveals a reasonable structure refinement. The displacement parameters of all atoms (silicon, carbon and oxygen) were refined anisotropically. The displacement parameters of the silicon atoms were constraint to be identical. The experimental crystallographic parameters at 6(1) GPa are in good agreement with the data derived from the DFT calculations at the same pressure: a = 4.3702 Å, b = 4.7845 Å, c = 7.4430 Å and β = 90.19° (V = 155.63 Å^3). The experimentally obtained lattice parameters ambient conditions are a = 4.378(6) Å, b = 5.094(2) Å, c = 7.387(1) Å and β = 90.8(2)° (V = 164.7(4) Å^3). They also agree with the values derived from the DFT calculations within the expected uncertainties (a = 4.4117 Å, b = 5.1857 Å, c = 7.4189 Å, β = 90.35° and V = 169.73 Å^3). The atomic coordinates for Si[CO_3]$_2$-$P2_1/n$ at 40(2) GPa are listed in Table S S2. The anisotropic displacement parameters of the silicon, carbon and the oxygen atoms can be found in the cif-file.

Table S2: Atomic coordinates and equivalent displacement parameters (Å^2) of Si[CO_3]$_2$-$P2_1/n$ at 40(2) GPa obtained by single crystal structure refinement.

**Atom**	**Site**	x	y	z	U^*_{eq}	$s.o.f.$
Si1	2d	0.0	0.5	0.5	0.0081(8)	0.225(6)
Si2	2b	0.0	1.0	0.5	0.0053(3)	0.770(7)
C1	4e	0.5040(7)	1.2540(4)	0.6652(2)	0.0065(5)	1.0
O1	4e	0.1973(5)	0.7519(3)	0.3437()	0.0087(5)	1.0
O2	4e	0.1371(5)	0.7670(3)	0.6794(2)	0.0083(4)	1.0
O3	4e	0.3347(5)	1.2440(3)	0.5192(2)	0.0092(5)	1.0

* U_{eq} is derived as 1/3 of the orthogonalized U_{ij} tensor

Raman spectroscopy on $Si[CO_3]_2$

We measured Raman spectroscopy on $Si[CO_3]_2$-$P2_1/n$ during decompression of the DAC. We did not observe significant changes in the experimental Raman spectrum other than a slight progressive deterioration of crystallinity during decompression. Fig. S2 b shows exemplary the experimental Raman spectrum of $Si[CO_3]_2$-$P2_1/n$ at 6(1) GPa in comparison to a theoretical spectrum derived from or DFT-based calculations. Our calculated structural model reproduces the experimental Raman spectrum very well. At this pressure phase I is the stable polymorph of CO_2. We could observe the characteristic Raman modes of CO_2-I (Fig. S2 a) in our experimental Raman spectrum of $Si[CO_3]_2$-$P2_1/n$ (Fig. S2 b).

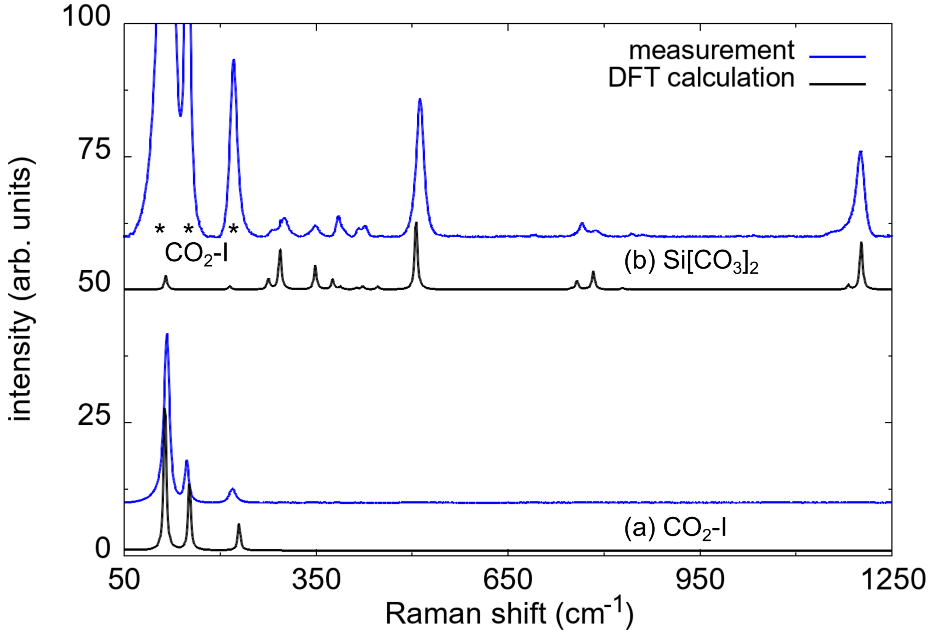

Figure S2: Raman spectroscopy at 6(1) GPa: (a) Raman spectra for CO_2-I. (b) Raman spectra for $Si[CO_3]_2$-$P2_1/n$. Experimental Raman spectra are shown in blue, DFT-based calculations are shown in black. The Raman shifts of the theoretical spectra were scaled by 2–4%. Peaks of CO_2-I in the Raman spectrum of $Si[CO_3]_2$-$P2_1/n$ are marked by an asterisk (∗).

In addition, we used the structural model of $Si[CO_3]_2$-$P2_1/n$ for our DFPT-calculations and calculated selected eigenvectors of the atomic displacements at 40 GPa. We found that the dominant Raman mode at $\approx$ 630 cm^{-1} (Fig. 2 c in the main text), belongs to a complex distortion of the $[SiO_6]$-octahedra (Fig. 2 e in the main text). In addition, an analysis of the polarization vector of the prominent Raman mode at $\approx$ 1250 cm^{-1} at 40 GPa (Fig. 2 c in the main text) shows that it is a pure $[CO_3]^{2-}$-stretching mode (Fig. S3).

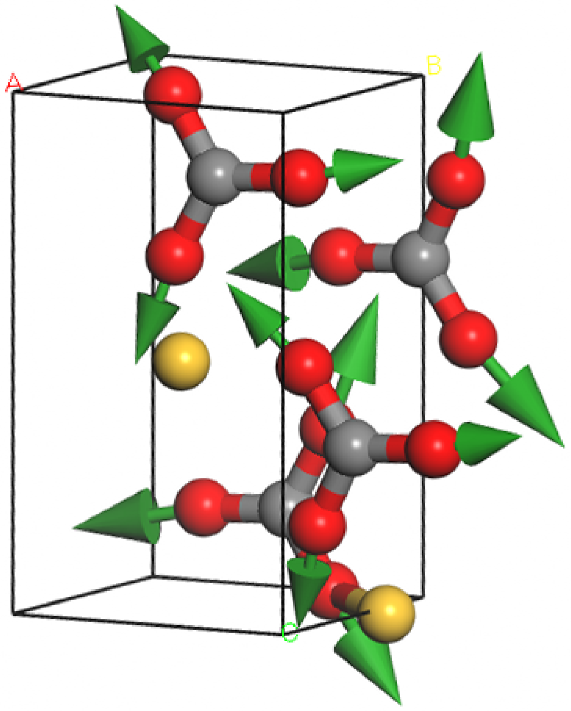

Figure S3: Eigenvector of the atomic displacements for the characteristic Raman mode of $Si[CO_3]_2$-$P2_1/n$ at $\approx$ 1250 cm^{-1} (40 GPa).

Compressibility of $Si[CO_3]_2$

The p, V relation for $Si[CO_3]_2$-$P2_1/n$ was derived from our DFT-based calculations in the pressure range between 0 GPa and 50 GPa. In order to obtain the bulk modulus (K_0) and its pressure derivative (K_p) we fitted a 3rd-order Vinet equation of states (EoS) to unit cell volume obtained by the calculations (Fig. S4) using the software package EOSFit7-GUI (*69, 70*). The theoretical bulk modulus of $Si[CO_3]_2$-$P2_1/n$ derived from the p, V relation is $K_0 = 53.1(4)$ GPa with $K_\mathrm{p} = 6.40(4)$. K_0 from the p, V relation is in agreement with the value derived from our stress-strain calculations (50.7(4) GPa).

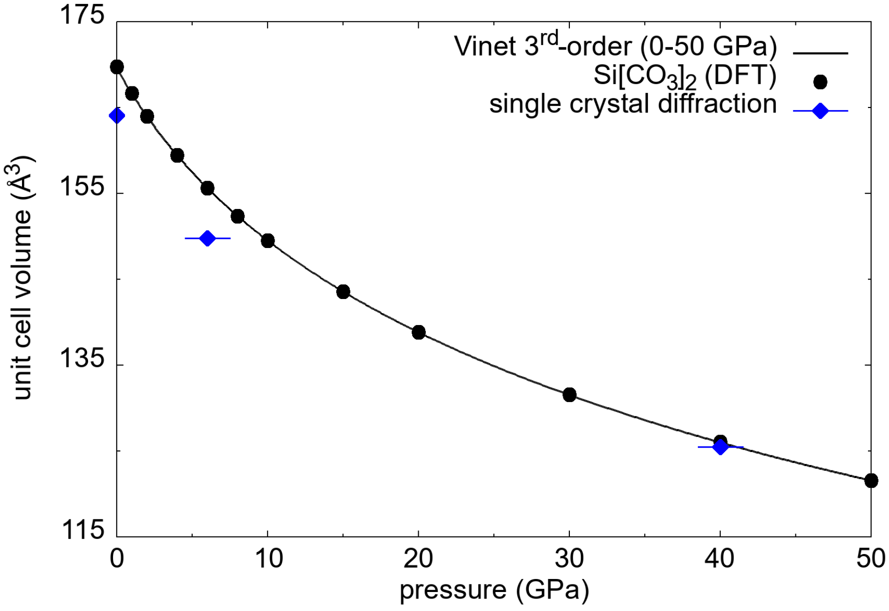

Figure S4: A Vinet EoS was fitted to the p, V-data of $Si[CO_3]_2$-$P2_1/n$ obtained by DFT-based calculations in the pressure range between 0 GPa and 50 GPa. The experimentally obtained unit cell volume from the single crystal data collection at 40(2) GPa, 6(1) GPa and at ambient conditions are shown for comparison.

Form the DFT-calculations we found that the compression behavior of $Si[CO_3]_2$-$P2_1/n$ is significantly anisotropic. The crystal structure is highly compressible along the b-axis, while it is incompressible along the a- and c-axes. Figure S5 shows the relative changes in the lattice parameters of $Si[CO_3]_2$-$P2_1/n$.

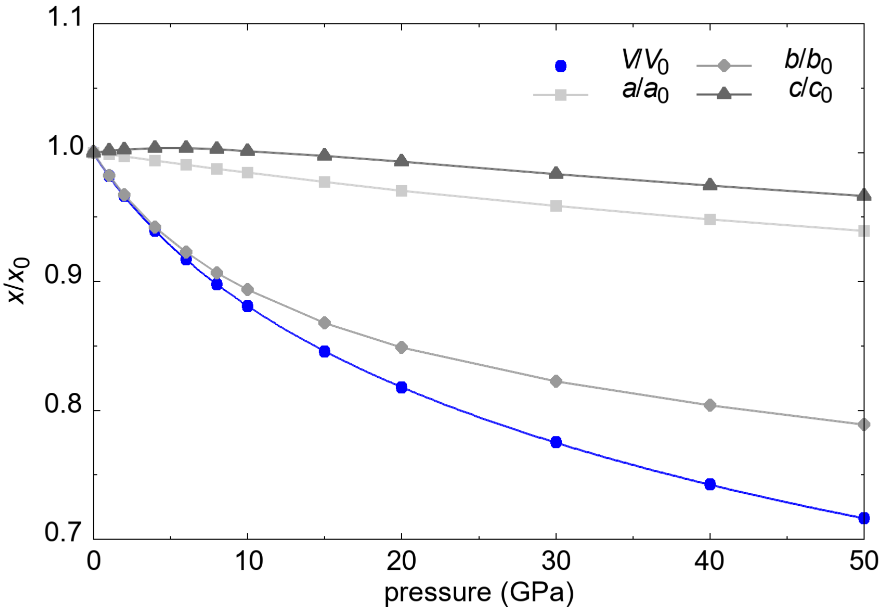

Figure S5: Relative changes in the a, b and c lattice parameters of $Si[CO_3]_2$-$P2_1/n$ derived from DFT-based calculations between 0 GPa and 50 GPa. The relative change in the unit cell volume together with the corresponding Vinet EoS fit (Fig. S4) is shown for comparison.

Elastic stiffness coefficients of Si[CO_3]$_2$

We computed the lastic stiffens coefficients (c_{ij}) for Si[CO_3]$_2$-$P2_1/n$ at 0 GPa by the stress strain-method. The tensor components from DFT-based calculations are listed in Table S3. The bulk modulus computed from the elastic stiffness tensor is 52(1) GPa. As all eigenvalues of the stiffness matrix are positive, the structure is stable with respect to small distortions.

Table S3: Elastic stiffness coefficients (c_{ij}) for Si[CO_3]$_2$-$P2_1/n$ at 0 GPa obtained by DFT-based strain-stress calculations.

c_{ij}	**DFT calculations (GPa)**
c_{11}	280.6(5)
c_{22}	56(1)
c_{33}	186(8)
c_{44}	73.9(5)
c_{55}	104.8(7)
c_{66}	37.3(3)
c_{12}	26.8(7)
c_{13}	18(2)
c_{15}	-15.9(5)
c_{23}	68.0(4)
c_{25}	4.3(9)
c_{35}	-4.1(9)
c_{46}	-0.6(2)

Comparison ofthe structures of $Be[CO_3]$ and $Si[CO_3]_2$

The crystal structure of $Si[CO_3]_2$-$P2_1/n$ characterized by SiO_6-octahedra. This structural units of the type $[M(CO_3)_6]^{n-}$ occur also in common carbonates such as such as calcite ($Ca[CO_3]$) or high-pressure Be-carbonate ($Be[CO_3]$) (*32, 37*). Fig. S6 shows a comparison how the MO_6-octahedra assemble into close-packed layers through shared carbonate groups in calcite-type $Be[CO_3]$ (AB-CABC sequence) and $Si[CO_3]_2$-$P2_1/n$ (AAA sequence).

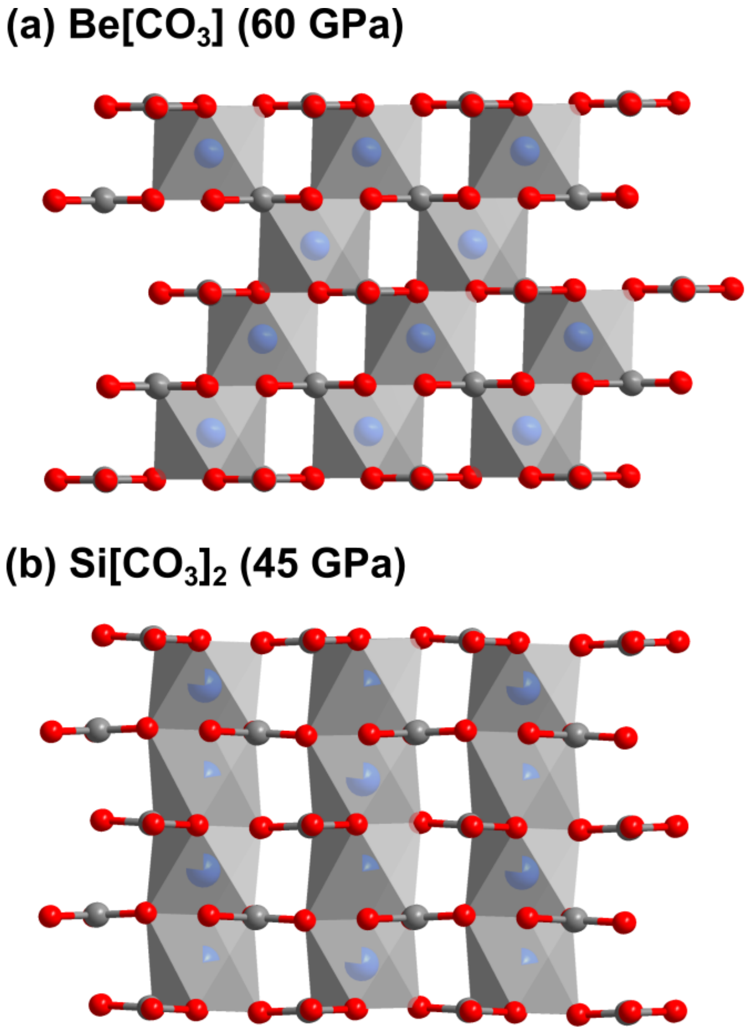

Figure S6: Close-packed layers of MO_6-octahedra coordinated by six $[CO_3]^{2-}$-groups: (a) In $Be[CO_3]$-$R\bar{3}c$ at 60(2) GPa. (*32*) (b) In $Si[CO_3]_2$-$P2_1/n$ at 40(2) GPa.

Reaction of cristobalite with CO_2

In order to understand if $Si[CO_3]_2$ can also be obtained from a SiO_2 precursor, we performed a second set of experiments. A cristobalite and CO_2-mixture was laser-heated at $\approx$ 40 GPa to a maximum temperature of T_{max} = 1800(200) K. After laser-heating we could identify the characteristic Raman signal of $Si[CO_3]_2$ in our experimental data, confirming that the reaction had occurred (Fig. S7).

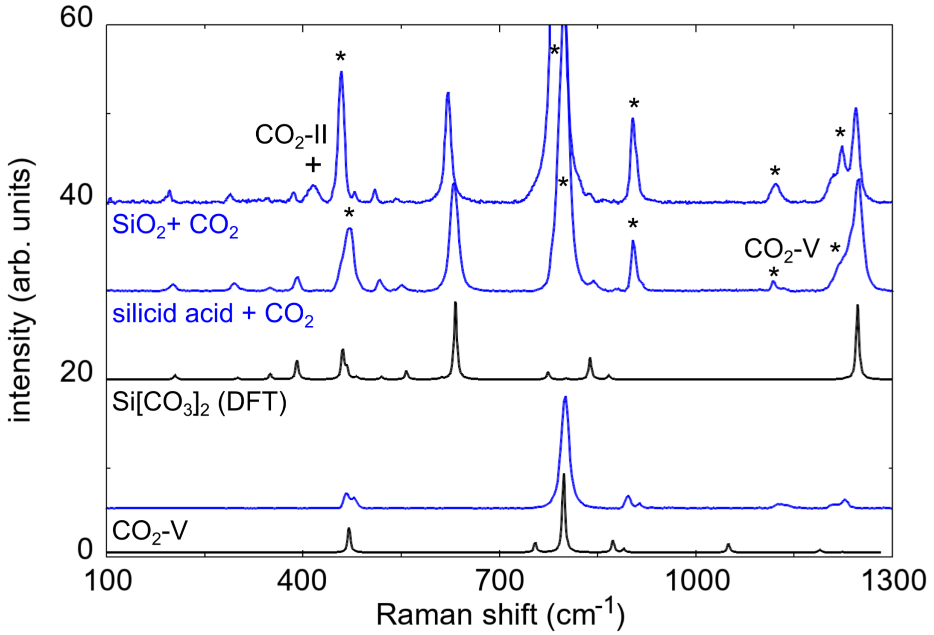

Figure S7: Raman spectroscopy of CO_2-V and $Si[CO_3]_2$ at 40(2) GPa after laser-heating a cristobalite + CO_2-mixture to T_{max} = 1800(200) K. Experimental Raman spectra are shown in blue, DFT-based calculations are shown in black. The Raman shifts of the theoretical spectra were scaled by 2–4%. Peaks of CO_2-V in the Raman spectrum of $Si[CO_3]_2$-$P2_1/n$ are marked by an asterisk (∗).

REFERENCES

1. R. J. Reeder, *Carbonates: Mineralogy and Chemistry* (De Gruyter, 1983).

2. N. R. McKenzie, B. K. Horton, S. E. Loomis, D. F. Stockli, N. J. Planavsky, C.-T. A. Lee, Continental arc volcanism as the principal driver of icehouse-greenhouse variability. *Science* **352**, 444–447 (2016).

3. M. M. Hirschmann, Comparative deep Earth volatile cycles: The case for C recycling from exosphere/mantle fractionation of major (H_2O, C, N) volatiles and from H_2O/Ce, CO_2/Ba, and CO_2/Nb exosphere ratios. *Earth Planet. Sci. Lett.* **502**, 262–273 (2018).

4. P. B. Kelemen, C. E. Manning, Reevaluating carbon fluxes in subduction zones, what goes down, mostly comes up. *Proc. Natl. Acad. Sci. U.S.A.* **11**, E3997–E4006 (2015).

5. P. D. Clift, A revised budget for Cenozoic sedimentary carbon subduction. *Rev. Geophys.* **55**, 97–125 (2017).

6. L. Bayarjargal, C.-J. Fruhner, N. Schrodt, B. Winkler, $CaCO_3$ phase diagram studied with Raman spectroscopy at pressures up to 50 GPa and high temperatures and DFT modeling. *Phys. Earth Planet. Inter.* **281**, 31–45 (2018).

7. J. Binck, S. Chariton, M. Stekiel, L. Bayarjargal, W. Morgenroth, V. Milman, L. Dubrovinsky, B. Winkler, High-pressure, high-temperature phase stability of iron-poor dolomite and the structures of dolomite-IIIc and dolomite-V. *Phys. Earth Planet. Inter.* **299**, 106403 (2020).

8. S. Kakizawa, T. Inoue, H. Suenami, T. Kikegawa, Decarbonation and melting in $MgCO_3$–SiO_2 system at high temperature and high pressure. *J. Mineral. Petrol. Sci.* **110**, 179–188 (2015).

9. X. Li, Z. Zhang, J.-F. Lin, H. Ni, V. B. Prakapenka, Z. Mao, New high-pressure phase of $CaCO_3$ at the topmost lower mantle: Implication for the deep-mantle carbon transportation. *Geophys. Res. Lett.* **45**, 1355–1360 (2018).

10. J. Gao, X. Wu, X. Yuan, W. Su, Fate of carbonates in the Earth's mantle (10-136 GPa). *Front. Earth Sci.* **10**, 837775 (2018).

11. D. Spahr, J. König, L. Bayarjargal, V. Milman, A. Perlov, H.-P. Liermann, B. Winkler, Sr[C_2O_5] is an inorganic pyrocarbonate salt with $[C_2O_5]^{2-}$ complex anions. *J. Am. Chem. Soc.* **144**, 2899–2904 (2022).

12. D. J. Spahr, L. Bayarjargal, M. Bykov, L. Brüning, T. H. Reuter, V. Milman, H.-P. Liermann, B. Winkler, High-pressure synthesis of acentric sodium pyrocarbonate, $Na_2[C_2O_5]$. *Dalton Trans.* **53**, 40–44 (2023).

13. D. Spahr, L. Bayarjargal, M. Bykov, L. Brüning, P. L. Jurzick, Y. Wang, V. Milman, K. Refson, M. Mezouar, B. Winkler, $Ca_3[C_2O_5]_2[CO_3]$ is a pyrocarbonate which can be formed at conditions prevalent in the Earth's transition zone. *Commun. Chem.* **7**, 238 (2024).

14. M. Santoro, F. Gorelli, J. Haines, O. Cambon, C. Levelut, G. Garbarino, Silicon carbonate phase formed from carbon dioxide and silica under pressure. *Proc. Natl. Acad. Sci. U.S.A.* **108**, 7689–7692 (2011).

15. M. Santoro, F. A. Gorelli, R. Bini, A. Salamat, G. Garbarino, C. Levelut, O. Cambon, J. Haines, Carbon enters silica forming a cristobalite-type CO_2–SiO_2 solid solution. *Nat. Commun.* **5**, 3761 (2014).

16. D. Santamaria-Perez, C. McGuire, A. Makhluf, A. Kavner, R. Chuliá-Jordan, J. L. Jorda, F. Rey, J. Pellicer-Porres, D. Martinez-Garcá, P. Rodriguez-Hernández, A. Muñoz, Strongly-driven Re + CO_2 redox reaction at high-pressure and high-temperature. *Nat. Commun.* **7**, 13647 (2016).

17. D. Santamaria-Perez, T. Marqueño, S. MacLeod, J. Ruiz-Fuertes, D. Daisenberger, R. Chuliá-Jordan, D. Errandonea, J. Luis Jordá, F. Rey, C. McGuire, A. Makhluf, A. Kavner, C. Popescu, Structural evolution of CO_2-filled pure silica LTA zeolite under high-pressure high-temperature conditions. *Chem. Mater.* **29**, 4502–4510 (2017).

18. A. Morales-García, M. Marqués, J. M. Menéndez, D. Santamaría-Pérez, V. G. Baonza, J. M. Recio, First-principles study of structure and stability in Si-C-O-based materials. *Theor. Chem. Acc.* **132**, 1308 (2013).

19. R. Zhou, B. Qu, J. Dai, X. C. Zeng, Unraveling crystalline structure of high-pressure phase of silicon carbonate. *Phys. Rev. X* **4**, 011030 (2014).

20. M. Marqués, A. Morales-García, J. M. Menéndez, V. G. Baonza, J. M. Recio, A novel crystalline SiCO compound. *Phys. Chem. Chem. Phys.* **17**, 25055–25060 (2015).

21. B. Qu, D. Li, L. Wang, J. Wu, R. Zhou, B. Zhang, X. C. Zeng, Mechanistic study of pressure and temperature dependent structural changes in reactive formation of silicon carbonate. *RSC Adv.* **6**, 26650–26657 (2016).

22. X. Yong, J. S. Tse, J. Chen, Mechanism of chemical reactions between SiO_2 and CO_2 under mantle conditions. *ACS Earth Space Chem.* **2**, 548–555 (2018).

23. A. M. Dziewonski, D. L. Anderson, Preliminary reference Earth model. *Phys. Earth Planet. Inter.* **25**, 297–356 (1981).

24. J. Ritsema, W. Xu, L. Stixrude, C. Lithgow-Bertelloni, Estimates of the transition zone temperature in a mechanically mixed upper mantle. *EPSL* **277**, 244–252 (2009).

25. T. Katsura, A. Yoneda, D. Yamazaki, T. Yoshino, E. Ito, Adiabatic temperature profile in the mantle. *Phys. Earth Planet. Inter.* **183**, 212–218 (2010).

26. P. Nimis, M. Alvaro, F. Nestola, R. J. Angel, K. Marquardt, G. Rustioni, J. W. Harris, F. Marone, First evidence of hydrous silicic fluid films around solid inclusions in gem-quality diamonds. *Lithos* **260**, 384–389 (2016).

27. D. Scelta, K. F. Dziubek, M. Ende, R. Miletich, M. Mezouar, G. Garbarino, R. Bini, Extending the stability field of polymeric carbon dioxide phase V beyond the Earth’s geotherm. *Phys. Rev. Lett.* **126**, 065701 (2021).

28. F. Datchi, B. Mallick, A. Salamat, S. Ninet, Structure of polymeric carbon dioxide CO_2-V. *Phys. Rev. Lett.* **108**, 125701 (2012).

29. K. Aoki, H. Yamawaki, M. Sakashita, Y. Gotoh, K. Takemura, Crystal structure of the high-pressure phase of solid CO_2. *Science* **263**, 356–358 (1994).

30. H. Olijnyk, A. P. Jephcoat, Vibrational studies on CO_2 up to 40 GPa by Raman spectroscopy at room temperature. *Phys. Rev. B* **57**, 879–888 (1998).

31. J. Binck, L. Bayarjargal, S. S. Lobanov, W. Morgenroth, R. Luchitskaia, C. J. Pickard, V. Milman, K. Refson, D. B. Jochym, P. Byrne, B. Winkler, Phase stabilities of $MgCO_3$ and $MgCO_3$-II studied by Raman spectroscopy, X-ray diffraction, and density functional theory calculations. *Phys. Rev. Mater.* **4**, 055001 (2020).

32. D. Spahr, L. Bayarjargal, E. Bykova, M. Bykov, L. Brüning, V. Kovalev, V. Milman, J. Wright, B. Winkler, 6-Fold-coordinated beryllium in calcite-type be Be[CO_3]. *Inorg. Chem.* **63**, 19513–19517 (2024).

33. N. Biedermann, S. Speziale, B. Winkler, H. J. Reichmann, M. Koch-Müller, G. Heide, High-pressure phase behavior of $SrCO_3$: An experimental and computational Raman scattering study. *Phys. Chem. Minerals* **44**, 335–343 (2017).

34. D. Spahr, L. Bayarjargal, E. Bykova, M. Bykov, T. H. Reuter, L. Brüning, P. L. Jurzick, L. Wedek, V. Milman, B. Wehinger, B. Winkler, Synthesis and crystal structure of acentric anhydrous beryllium carbonate Be(CO_3). *Chem. Commun.* **60**, 10208–10211 (2024).

35. G. Fiquet, F. Guyot, M. Kunz, J. Matas, D. Andrault, M. Hanfland, Structural refinements of magnesite at very high pressure. *Am. Mineral.* **87**, 1261–1265 (2002).

36. Y. Zhang, S. Chariton, J. He, S. Fu, F. Xu, V. B. Prakapenka, J.-F. Lin, Atomistic insight into the ferroelastic post-stishovite transition by high-pressure single-crystal X-ray diffraction. *Am. Mineral.* **108**, 110–119 (2023).

37. H. Effenberger, K. Mereiter, J. Zemann, Crystal structure refinements of magnesite, calcite, rhodochrosite, siderite, smithonite, and dolomite, with discussion of some aspects of the stereochemistry of calcite type carbonates. *Z. Kristallogr.* **156**, 233–243 (1981).

38. L. Bayarjargal, D. Spahr, V. Milman, J. Marquardt, N. Giordano, B. Winkler, Anhydrous aluminium carbonates and isostructural compounds. *Inorg. Chem.* **62**, 13910–13918 (2023).

39. R. D. Shannon, Revised effective ionic radii and systematic studies of interatomic distances in halides and chalcogenides. *Acta Crystallogr.* **32**, 751–767 (1976).

40. A. H. Davis, N. V. Solomatova, R. Caracas, A. J. Campbell, Carbon storage in Earth's deep interior implied by carbonate-silicate-iron melt miscibility. *Geochem. Geophys. Geosyst.* **24**, e2023GC010896 (2023).

41. D. Spahr, L. Bayarjargal, E. Bykova, M. Bykov, G. L. Murphy, P. Kegler, V. Milman, N. Giordano, B. Winkler, High-pressure synthesis of $U_2[CO_3]_3$ and $U[CO_3]_2$ as potential host phases for uranium in the Earth's mantle. *Commun. Chem.* **9**, 112 (2026).

42. J. Zhang, R. J. Reeder, Comparative compressibilities of calcite-structure carbonates: Deviations from empirical relations. *Am. Mineral.* **84**, 861–870 (1999).

43. R. Boehler, New diamond cell for single–crystal X-ray diffraction. *Rev. Sci. Instrum.* **77**, 115103 (2006).

44. X. Li, X. Yin, L. Zhang, S. He, The devitrification kinetics of silica powder heat-treated in different conditions. *J. Non Cryst. Solids* **354**, 3254–3259 (2008).

45. D. Spahr, L. Bayarjargal, L. Brüning, V. Kovalev, E. Bykova, M. Bykov, V. Milman, M. Mezouar, B. Winkler, Synthesis and crystal structure of anhydrous di-iodyl carbonate $(IO_2)_2[CO_3]$, hosting I^{5+}-cations. *JACS Au* **5**, 4675–4680 (2025).

46. Y. Akahama, H. Kawamura, Pressure calibration of diamond anvil Raman gauge to 310 GPa. *J. Appl. Phys.* **100**, 043516 (2006).

47. H.-P. Liermann, Z. Konôpková, W. Morgenroth, K. Glazyrin, J. Bednarčik, E. E. McBride, S. Petitgirard, J. T. Delitz, M. Wendt, Y. Bican, A. Ehnes, I. Schwark, A. Rothkirch, M. Tischer, J. Heuer, H. Schulte-Schrepping, T. Kracht, H. Franz, The extreme conditions beamline P02.2 and

the extreme conditions science infrastructure at PETRAIII. *J. Synchrotron Radiat.* **22**, 908–924 (2015).

48. J. Wright, C. Giacobbe, M. Majku, New opportunities at the materials science beamline at ESRF to exploit high energy nano-focus X-ray beams. *Curr. Opin. Solid. St. M.* **24**, 100818 (2020).

49. O. V. Dolomanov, L. J. Bourhis, R. J. Gildea, J. A. K. Howard, H. Puschmann, *OLEX2*: A complete structure solution, refinement and analysis program. *J. Appl. Cryst.* **42**, 339–341 (2009).

50. G. M. Sheldrick, *SHELXT*—Integrated space-group and crystal-structure determination. *Acta Crystallogr.* **71**, 3–8 (2015).

51. G. M. Sheldrick, Crystal structure refinement with *SHELXL*. *Acta Crystallogr.* **71**, 3–8 (2015).

52. P. Hohenberg, W. Kohn, Inhomogeneous electron gas. *Phys. Rev.* **136**, B864–B871 (1964).

53. J. P. Perdew, K. Burke, M. Ernzerhof, Generalized gradient approximation made simple. *Phys. Rev. Lett.* **77**, 3865–3868 (1996).

54. S. J. Clark, M. D. Segall, C. J. Pickard, P. J. Hasnip, M. I. J. Probert, K. Refson, M. C. Payne, First principles methods using CASTEP. *Z. Kristallogr.* **220**, 567–570 (2005).

55. BIOVIA, Materials Studio, San Diego, USA (2025).

56. A. Tkatchenko, M. Scheffler, Accurate molecular van der Waals interactions from ground-state electron density and free-atom reference data. *Phys. Rev. Lett.* **102**, 073005 (2009).

57. S. Baroni, S. de Gironcoli, A. Dal Corso, P. Giannozzi, Phonons and related crystal properties from density-functional perturbation theory. *Rev. Mod. Phys.* **73**, 515–562 (2001).

58. K. Refson, P. R. Tulip, S. J. Clark, Variational density-functional perturbation theory for dielectrics and lattice dynamics. *Phys. Rev. B* **73**, 155114 (2006).

59. K. Miwa, Prediction of Raman spectra with ultrasoft pseudopotentials. *Phys. Rev. B* **84**, 094304 (2011).

60. C. S. Yoo, H. Cynn, F. Gygi, G. Galli, V. Iota, M. Nicol, S. Carlson, D. Häusermann, C. Mailhiot, Crystal structure of carbon dioxide at high pressure: "Superhard" polymeric carbon dioxide. *Phys. Rev. Lett.* **83**, 5527–5530 (1999).

61. L. R. Benedetti, P. Loubeyre, Temperature gradients, wavelength-dependent emissivity, and accuracy of high and very-high temperatures measured in the laser-heated diamond cell. *High Press. Res.* **24**, 423–445 (2004).

62. Z. Du, G. Amulele, L. R. Benedetti, K. K. M. Lee, Mapping temperatures and temperature gradients during flash heating in a diamond-anvil cell. *Rev. Sci. Instrum.* **84**, 075111 (2013).

63. M. Wojdyr, *Fityk*: A general-purpose peak fitting program. *J. Appl. Cryst.* **43**, 1126–1128 (2010).

64. C. Prescher, V. B. Prakapenka, *DIOPTAS*: A program for reduction of two-dimensional X-ray diffraction data and data exploration. *High. Press. Res.* **35**, 223–230 (2015).

65. Agilent, CrysAlis PRO, Yarnton, England (2014).

66. A. Aslandukov, M. Aslandukov, N. Dubrovinskaia, L. Dubrovinsky, *Domain Auto Finder* (*DAFi*) program: The analysis of single-crystal X-ray diffraction data from polycrystalline samples. *J. Appl. Cryst.* **55**, 1383–1391 (2022).

67. K. Lejaeghere, G. Bihlmayer, T. Björkman, P. Blaha, S. Blügel, V. Blum, D. Caliste, I. E. Castelli, S. J. Clark, A. Dal Corso, S. de Gironcoli, T. Deutsch, J. K. Dewhurst, I. Di Marco, C. Draxl, M. Dułak, O. Eriksson, J. A. Flores-Livas, K. F. Garrity, L. Genovese, P. Giannozzi, M. Giantomassi, S. Goedecker, X. Gonze, O. Granas, E. K. U. Gross, A. Gulans, F. Gygi, D. R. Hamann, P. J. Hasnip, N. A. W. Holzwarth, D. Iusan, D. B. Jochym, F. Jollet, D. Jones, G. Kresse, K. Koepernik, E. Kücükbenli, Y. O. Kvashnin, I. L. M. Locht, S. Lubeck, M. Marsman, N. Marzari, U. Nitzsche, L. Nordström, T. Ozaki, L. Paulatto, C. J. Pickard, W. Poelmans, M. I. J. Probert, K. Refson, M. Richter, G.-M. Rignanese, S. Saha, M. Scheffler, M. Schlipf, K.

Schwarz, S. Sharma, F. Tavazza, P. Thunström, A. Tkatchenko, M. Torrent, D. Vanderbilt, M. J. van Setten, V. Van Speybroeck, J. M. Wills, J. R. Yates, G.-O. Zhang, S. Cottenier, Reproducibility in density functional theory calculations of solids. *Science* **351**, aad3000 (2016).

68. H. J. Monkhorst, J. D. Pack, Special points for Brillouin-zone integrations. *Phys. Rev. B* **13**, 5188–5192 (1976).

69. P. Vinet, J. H. Rose, J. Ferrante, J. R. Smith, Universal features of the equation of state of solids. *Phys. Condens. Matter* **1**, 1941–1963 (1989).

70. J. Gonzalez-Platas, M. Alvaro, F. Nestola, R. Angel, *EosFit7-GUI*: A new graphical user interface for equation of state calculations, analyses and teaching. *J. Appl. Cryst.* **49**, 1377–1382 (2016).
